# Endocrine cybernetics: neuropeptides as molecular switches in behavioural decisions

**DOI:** 10.1098/rsob.220174

**Published:** 2022-07-27

**Authors:** Dick R. Nässel, Meet Zandawala

**Affiliations:** ^1^ Department of Zoology, Stockholm University, 10691 Stockholm, Sweden; ^2^ Neurobiology and Genetics, Theodor-Boveri-Institute, Biocenter, University of Würzburg, Am Hubland Würzburg 97074, Germany

**Keywords:** *Drosophila melanogaster*, neuromodulation, brain circuits, interneurons, peptide hormones

## Abstract

Plasticity in animal behaviour relies on the ability to integrate external and internal cues from the changing environment and hence modulate activity in synaptic circuits of the brain. This context-dependent neuromodulation is largely based on non-synaptic signalling with neuropeptides. Here, we describe select peptidergic systems in the *Drosophila* brain that act at different levels of a hierarchy to modulate behaviour and associated physiology. These systems modulate circuits in brain regions, such as the central complex and the mushroom bodies, which supervise specific behaviours. At the top level of the hierarchy there are small numbers of large peptidergic neurons that arborize widely in multiple areas of the brain to orchestrate or modulate global activity in a state and context-dependent manner. At the bottom level local peptidergic neurons provide executive neuromodulation of sensory gain and intrinsically in restricted parts of specific neuronal circuits. The orchestrating neurons receive interoceptive signals that mediate energy and sleep homeostasis, metabolic state and circadian timing, as well as external cues that affect food search, aggression or mating. Some of these cues can be triggers of conflicting behaviours such as mating versus aggression, or sleep versus feeding, and peptidergic neurons participate in circuits, enabling behaviour choices and switches.

## Graphical abstract

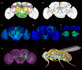

## 1. Introduction

Animal behaviour is plastic and dependent on the ability to modulate the hardwired neuronal circuitry by integrating cues from the external environment and internal states. These signals modify neuronal activity in multiple ways. They lead to relevant context-dependent changes in circuit properties and thereby add flexibility to behaviour and aids in decision-making (see [1–7]). Thus, internal conditions like for instance circadian time, metabolic state, reproductive drive and sleep homeostasis, as well as external cues such as temperature, light conditions and signals that trigger food search, aggression or mating, affect circuit activity in the brain, and hence behaviour. These internal and external signals can trigger conflicting behaviours, such as mating versus aggression, and need to be weighed to ensure a relevant outcome [2,5,7–13]. Furthermore, these states interact so that for example hunger lowers the threshold for aggression or increases it for sleep, and alterations in metabolic state affect reproductive behaviour [4,14–17].

Many of the known neuronal systems in *Drosophila* that modulate behavioural circuits in a state-dependent fashion and form switches between behaviours use different neuropeptides or biogenic amines such as serotonin, dopamine or octopamine (see [2,6,8,18–23]). These modulatory circuits or pathways are not necessarily hardwired, but rather they commonly depend on paracrine signalling or volume transmission, which is based on non-synaptic release of an amine or neuropeptide over a shorter or longer distance within the CNS [6,18,22,24–26]. Some of the modulatory signalling is even hormonal, via the circulation, representing interorgan communication (see for instance [18,27–32]).

This review discusses functional roles of neuropeptides and peptide hormones in different layers of neuromodulation that integrate internal and external stimuli to generate appropriate behaviour. Importantly, behaviour is also modified by experience and thus neuromodulation is important in learning and memory (see [12,33,34]). Some of the neuromodulatory systems that affect behaviour can concomitantly modulate the physiology of the organism thereby ensuring appropriate energy allocation, metabolic and ionic homeostasis as well as timing of activity and sleep rhythms. We review data that suggest that neuronal systems that use neuropeptides are hierarchically organized. At the top level large peptidergic neurons that arborize in multiple areas of the brain orchestrate or modulate global activity, and at the local level smaller neurons release peptides in restricted parts of neuronal circuits to provide executive neuromodulation (see [35]). At the executive level, where neuromodulation affects synaptic connections locally, it is common that neuropeptides act together with small molecule neurotransmitters (SMNs) (see [36–38]). Additionally, circulating peptide hormones may also act as organizers of behaviour and physiology at a high level in the hierarchy (see [18,27,29,30,39]). Some of these hormones are released from enteroendocrine cells (EECs) of the intestine [32,40–43]. Many of the EECs are nutrient-sensing and thus serve to signal the nutritional state of the organism to the brain, thereby modulating relevant nutrient/energy-dependent behaviours as well as regulating metabolic homeostasis [39,43–45]. Taken together, studies on *Drosophila* neuropeptides show that they are critical players in the coordination of the external and internal milieu to establish physiological homeostasis and to guide relevant behaviours. They do so by modulating the gain of sensory inputs and activity in circuits controlling behavioural output, including partaking in switches between conflicting behaviours and modulation of motor output.

## 2. Peptides in synaptic, paracrine and hormonal signalling

Peptides hold a special place in the pantheon of signalling substances in animals. They exist in numerous forms, which are structurally and functionally very diverse, and they are known to play multiple roles in developmental processes, as well as in regulation of most aspects of physiology and behaviour [6,18,30,46–50]. Peptides are evolutionarily ancient and are used for signalling also in organisms that lack a nervous system [51–53]. They are encoded by genes that give rise to precursor proteins from which the mature peptides are enzymatically cleaved (see [50,54–56]). In animals with more evolved nervous systems, peptides are produced by neurons, neurosecretory cells, endocrine cells and other cell types in various tissues and can act as neuromodulators, circulating hormones, cytokines and even as allomones for inter-individual signalling (e.g. sex peptide in *Drosophila*) and as toxins (reviewed in [18,30,47,50,57–60]). Here, we shall primarily discuss neuropeptides and peptide hormones produced by neurons and endocrine cells. The distinction between neuropeptides and peptide hormones used here is that neuropeptides are released to act on target cells within the CNS, or for short range action on muscles, glands or other peripheral tissues, whereas peptide hormones are released into the circulation for global action.

While hormonal roles of peptides are rather straightforward to investigate, it is more difficult to functionally delineate signalling pathways within the CNS that use neuropeptides. This is due to the fact that neuropeptides are commonly released non-synaptically and thus they partly disregard the hardwired synaptic circuitry and act at a larger distance than SMNs [25,36,38,57,58,61–63]. There are probably exceptions where neuropeptide release may also occur adjacent to regular synapses (localized extrasynaptic or parasynaptic release) (see [58,61]). The non-synaptic and diffuse neurotransmission is referred to as *paracrine signalling*, or volume transmission [63,64]. This type of diffuse signalling was already described in the pancreas in 1938 by Friedrich Feyrter (see [63,65,66]). Paracrine signalling is commonly superimposed on regular synaptic transmission with SMNs, where the neuropeptide plays a modulatory role, clearly seen when peptides act as co-transmitters at synapses [36–38,58,67–69]. Neuromodulation in a circuit can occur in two major modes: (i) extrinsic neuromodulation (peptidergic neurons interconnecting circuits) or (ii) intrinsic neuromodulation (peptidergic neurons that are part of the modulated circuit) [37,70,71]. In either case the direct targets of the neuropeptide action are difficult to resolve with standard molecular genetics techniques and establishment of regular neuronal connectomics may not necessary predict peptidergic ‘circuits' (see [22,72]).

## 3. Hierarchical organization of peptidergic neuromodulatory systems

Analysis of the neuronal connectome in *Drosophila* has provided anatomical maps of neurons and their chemical synapses forming circuits in major centres of the brain (see [73–76]). Onto these anatomical circuit maps we need to superimpose neuromodulatory neurons to better understand functional plasticity (see [72]). In the visual system of fast-moving insects, information processing is rapid and likely dependent to a large degree on fast synaptic signalling with SMNs. Thus, the visual connectome provides a more realistic dataset for modelling function [76–78]. Although neuromodulation also occurs in the visual system (see [79,80]), we ignore this system here. Instead, this review focuses on other parts of the brain such as for instance the olfactory and gustatory systems, the central complex, mushroom bodies, clock circuits and the pars intercerebralis, where neuromodulation is much more prominent and has been more intensely studied. First, we shall look at the organization of neuronal systems that use neuropeptides and peptide hormones.

**Figure 1.**
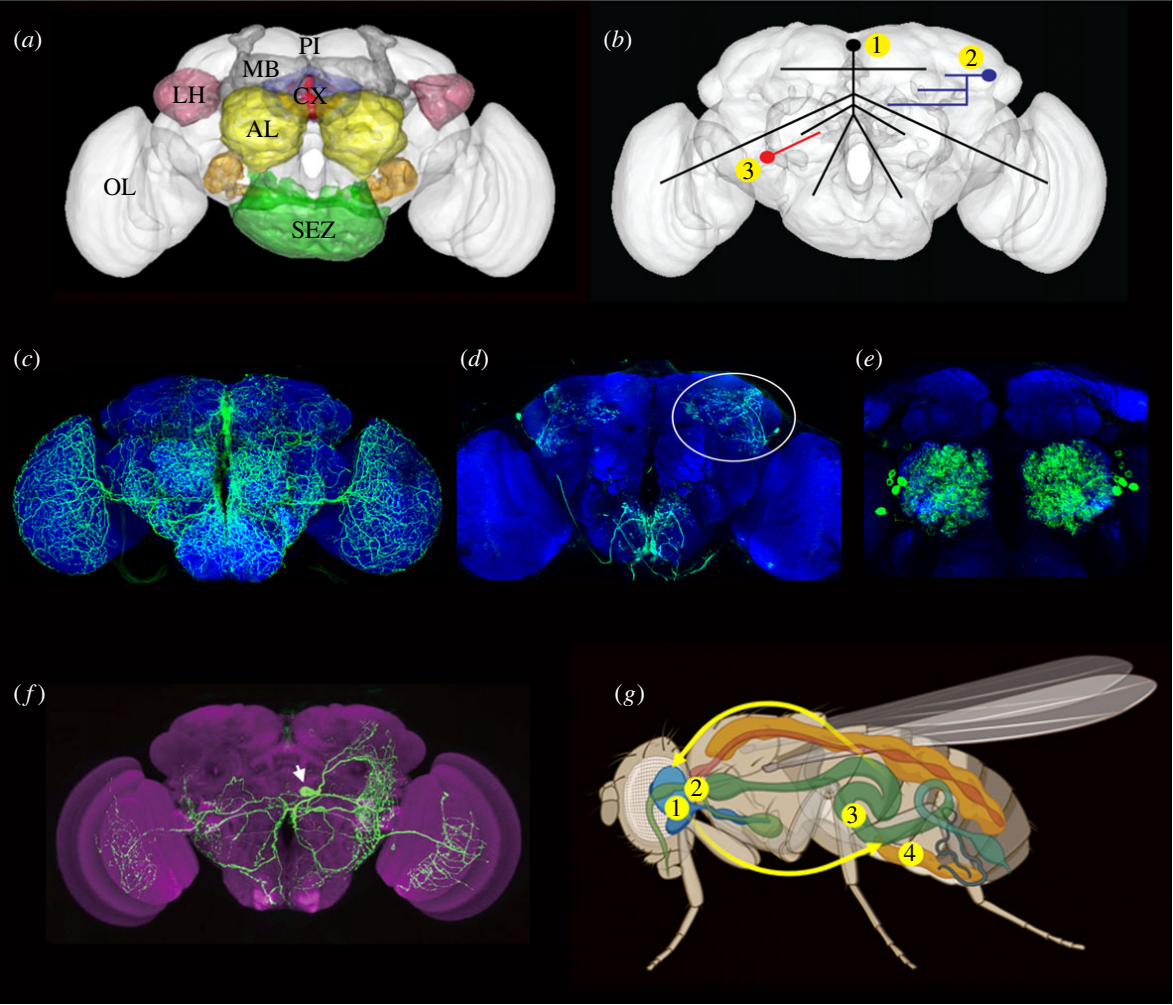
The *Drosophila* brain and hierarchical organization of peptidergic signalling. (*a*) The brain of adult *Drosophila* with some of the neuropils indicated in colour. The central complex (CX) is highlighted in blue and red. Pars intercerebralis (PI), optic lobe (OL), mushroom bodies (MB), antennal lobes (AL), lateral horn (LH) and suboesophageal zone (SEZ) are also highlighted. (*b*) Three levels of modulatory peptide signalling. (1) Widely arborizing orchestrating neurons innervate many neuropils and receive multiple inputs of different kinds. (2) Intermediate size peptidergic neurons (often in one hemisphere only) interconnect several regions. They may for instance receive inputs from clock neurons and others and supply outputs to the fan-shaped body to mediate nutrition-dependent sleep activity. (3) Local neurons mediate executive (intrinsic) neuromodulation, here exemplified by a local neuron in the antennal lobe. Such neurons can serve as an interface between different odour channels and also receive higher-level modulatory inputs. At this level also some of the odorant-sensitive neurons are known to signal locally with neuropeptides to modulate sensory gain. (*c–f*) Confocal images of GFP labelled neurons of the types indicated in (*b*). (*c*) SIFamide-expressing neurons widely arborizing throughout the brain (level 1 in (*b*)). (*d*) Leucokinin-expressing neurons. One of the LHLK neurons is encircled (level 2 in (*b*)). (*e*) A small number of local neurons (LNs) innervating each of the antennal lobes (level 3 in (*b*)). Several such neurons express tachykinin. (*f*) A single drosulfakinin (DSK)-expressing neurons (MP1a type) with wide branches (level 1 in (*b*)). (*g*) Interorgan signalling. Neurosecretory cells in the brain (1) and endocrine cells in corpora cardiaca (2) release hormones into the circulation that act on peripheral targets such as the intestine (3). The intestine and the fat body (4) produce peptide hormones that signal to the brain. Brains in (*a*) and (*b*) were made in RStudio using the Natverse package and (*g*) was generated in BioRender.

Each neuropeptide and peptide hormone has its own unique expression pattern. They are expressed in different types of neurons and/or neurosecretory cells in the CNS, as well as in cells of other tissues (see [18,81–83]). More specifically, peptides are produced by interneurons, sensory cells, motoneurons, neurosecretory cells, gut EECs and in some cases in glial cells [see [18]]. Here we deal primarily with peptides produced by sensory neurons, interneurons, neurosecretory cells, and intestinal EECs. The general organization of the *Drosophila* brain, types of peptidergic neurons and a scheme of interorgan communication are shown in figure 1*a–g*.

**Figure 2.**
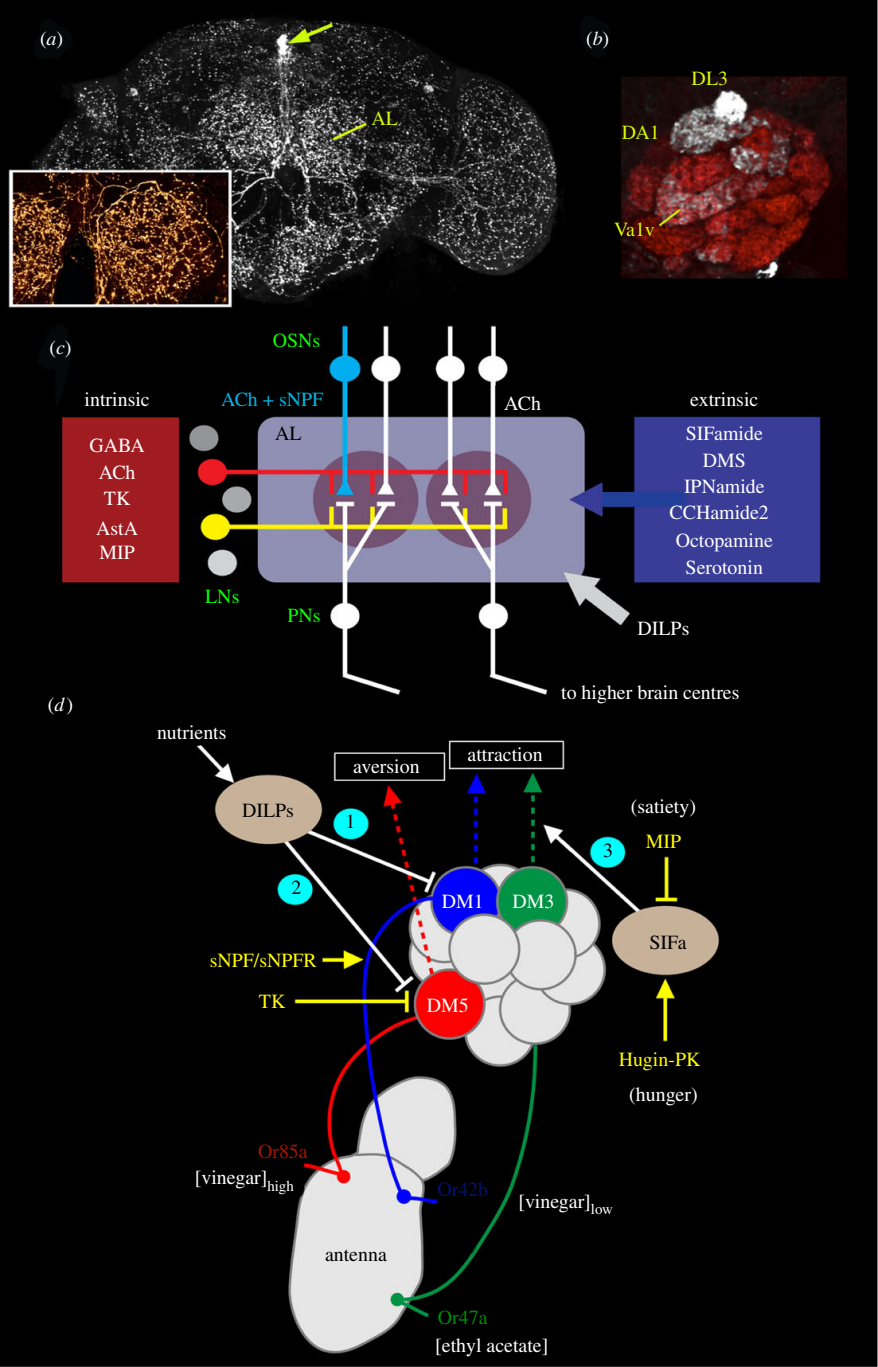
Different levels of peptidergic neuromodulation exemplified in odour signal processing. This figure illustrates two main levels of neuromodulation, orchestrating (extrinsic) and executive (local). (*a*) Orchestrating neurons expressing the neuropeptide SIFamide have widely arborizing processes in most regions of the *Drosophila* brain including the antennal lobe (AL). Four neuronal cell bodies (at arrow) give rise to the processes. The inset shows branches in the antennal lobe. (*b*) Localized, executive peptide signalling occurs in select glomeruli in the antennal lobe by short neuropeptide F (sNPF) produced in olfactory sensory neurons (OSNs) innervating the glomeruli. Three glomeruli innervated by sNPF-producing OSNs are seen here (DL3, DA1 and Va1v). The local sNPF-mediated modulation is described in the text. (*c*) Neurotransmitters and neuropeptides involved in intrinsic and extrinsic neuromodulation in the antennal lobe. Two glomeruli are shown here with OSNs and projection neurons (PNs). The substances used for intrinsic modulation are produced by local neurons (LNs) and some OSNs (light blue). Substances used for extrinsic modulation are produced by various types of large neurons that originate outside the olfactory system. Two LNs are highlighted in red and yellow. In addition, insulin-like peptides (DILPs) act presynaptically on OSNs via the circulation. All the OSNs use acetylcholine, but some subpopulations of the OSNs additionally use sNPF or myoinhibitory peptide (not shown). The PNs are also cholinergic and some of these co-express sNPF or TK. (*d*) This figure shows different levels of modulation of olfactory signals related to food odour attraction and aversion. Three channels responding to food odours from receptor (Or) to antennal lobe glomerulus (DM) are shown (red, blue and green). Two are modulated by nutrient-dependent hormonal DILP signals (1 and 2), and a third by orchestrating SIFamide (SIFa) neurons (3). These represent orchestrating signals. At the executive level, the Or42b receptor expressing OSNs also express sNPF and the sNPF receptor (sNPFR). In hungry flies, low levels of DILPs upregulate sNPF expression (1) and thus the signal strength to higher order olfactory neurons increases and food search increases (attraction). TK released from local neurons inactivates synaptic activity in Or85a neurons, and low levels of DILPs (in hungry flies) inhibit TK action (2) leading to aversion for high concentrations of food odour (vinegar). SIFa neurons modulate food attraction via Or47a-DM3 at the level of projection neurons (3) and are under regulation of myoinhibitory peptide (MIP) and Hugin-pyrokinin (Hugin-PK). (*a*) and (*b*) are altered from Carlsson *et al.* [85], (*c*) is redrawn from Nässel [36] which was based on an idea from Lizbinski & Dacks [86] and (*d*) is altered from Nässel *et al.* [35] which in turn was based on Sayin *et al.* [10].

Neuropeptide signalling acts at different levels of the neuronal circuitry [24,37,47,84] (figure 1*b*, table 1). Interneuronal peptides act in three main modes, correlated with the type of neurons they are expressed in (see [35]) (figure 1*c–f*). These modes are (i) *orchestrating signalling* (commonly state-dependent), (ii) *context-specific signalling* and (iii) *circuit-specific executive signalling*. Of these, 1 and 2 mediate extrinsic neuromodulation and 3 mainly intrinsic (figure 2*a–d*, table 1). In general, it is thought that these three main modes of signalling are part of a hierarchy with mode 1 at the top and mode 3 at the bottom level. Also, the temporal aspects of modulatory action (speed of onset and duration of modulation) appear to follow the same pattern with mode 1 being slowest and most sustained, whereas mode 3 is the fastest. Note that many given neuropeptides can act pleiotropically and thus have diverse roles at different levels of the hierarchy and this will be clarified in the text when appropriate. Clearly, the borders between these three levels/modes of neuropeptide action are not always distinct. Some peptidergic neurons may be hard to categorize (especially modes 2 and 3) and to an extent functions may be part of a spectrum rather than being distinct. Roughly speaking, the modes 1–3 neurons can be distinguished by the number of target neurons and the volume of brain being innervated by each neuron (figure 1*b*). The peptidergic neurons underlying the three modes of modulation interact with different synaptic circuits (and other modulatory circuits) and receive direct or indirect inputs mediating cues from the external environment and internal states. The three levels of peptidergic neuromodulation are outlined briefly below, and we will discuss further features in detail in later sections.

**Table 1.** Modes of neuromodulation, from local to global.

type of neuromodulation^a^	neuropeptide^b^	features^c^
executive signalling (local)	TK, sNPF	small neurons (restricted branching)
		acts in specific circuits
		local neuromodulation (fast)
		can act as co-transmitter
		sensory gain control
context-specific signalling (intermediate)	LK, AstA	intermediate sized neurons
		regulates several circuits
		context-specific inputs
		affects behavioural choices
orchestrating signalling (global)	SIFa, DSK	widely branching neurons
		global regulation (slow, sustained)
		multiple inputs and targets
		state- and context-dependent
		affects behavioural choices
		orchestrates behaviour
hormonal orchestrating signalling (global)	DILPs, AKH	IPCs, APCs^d^
		acts via circulation (slow, sustained)
		interorgan signalling
		nutrition-dependent
		orchestrates behaviour
		regulates physiology
		DILPs can affect GPCR expression^e^

^a^Refers to the modes of neuromodulation discussed in this review.

^b^These are only the neuropeptides discussed in this review, so not comprehensive.

^c^Main characteristics of signalling. The fast, slow and sustained refers to temporal aspects of modulation/action.

^d^IPCs, brain insulin-producing cells; APCs, AKH-producing cells in corpora cardiaca.

^e^DILP signalling regulates expression of sNPF and TK receptors (GPCRs) in the olfactory system and thereby affects executive signalling at the first synapse between olfactory sensory neurons and interneurons (gain control).

### 3.1. Orchestrating signalling using a few large-field interneurons

At the top level, internal state- or context-dependent signals can be integrated into orchestrating peptidergic systems that act widely in the brain. This type of system is commonly formed by a few, widely arborizing neurons that integrate multiple inputs (encoding external and internal cues) and convey basal states (e.g. metabolic status or different kinds of arousal) across many circuits (figures 1*c* and 2*a*). Signals that convey global states may feed into the more dynamic context-specific modulatory circuits, but can also set thresholds at the executive modulatory level (figure 2*b–d*). Thus, orchestrating neurons interact with other peptidergic systems at all levels. This kind of large peptidergic neurons can be part of circuits that weigh sensory inputs and internal states and hence form switches between competing behaviours (e.g. feeding and sleep, or mating and aggression). Examples of neuropeptides in this kind of neuronal circuit in *Drosophila* are SIFamide (SIFa) and drosulfakinin (DSK) [87–90].

### 3.2. Context-specific signalling

Some neuropeptides are produced in large neurons interconnecting several brain regions, but with fewer and less wide processes than the orchestrating neurons (figure 1*b*,*d*). These peptidergic neurons can mediate a context-specific influence (e.g. satiety or hunger, circadian timing and sleep homeostasis) in circuits at several locations. This type of peptidergic signalling can also recruit executive modulation within individual brain compartments. Examples of neuropeptides that are used in context-specific signalling in *Drosophila* are allatostatin A (AstA), and leucokinin (LK) (see e.g. [91–94]). Note that these peptides are not exclusively used by large interneurons, but also by neurosecretory cells (LK), or small interneurons and EECs (AstA) [91,95,96].

### 3.3. Circuit-specific executive signalling using multiple small field interneurons

**Figure 3.**
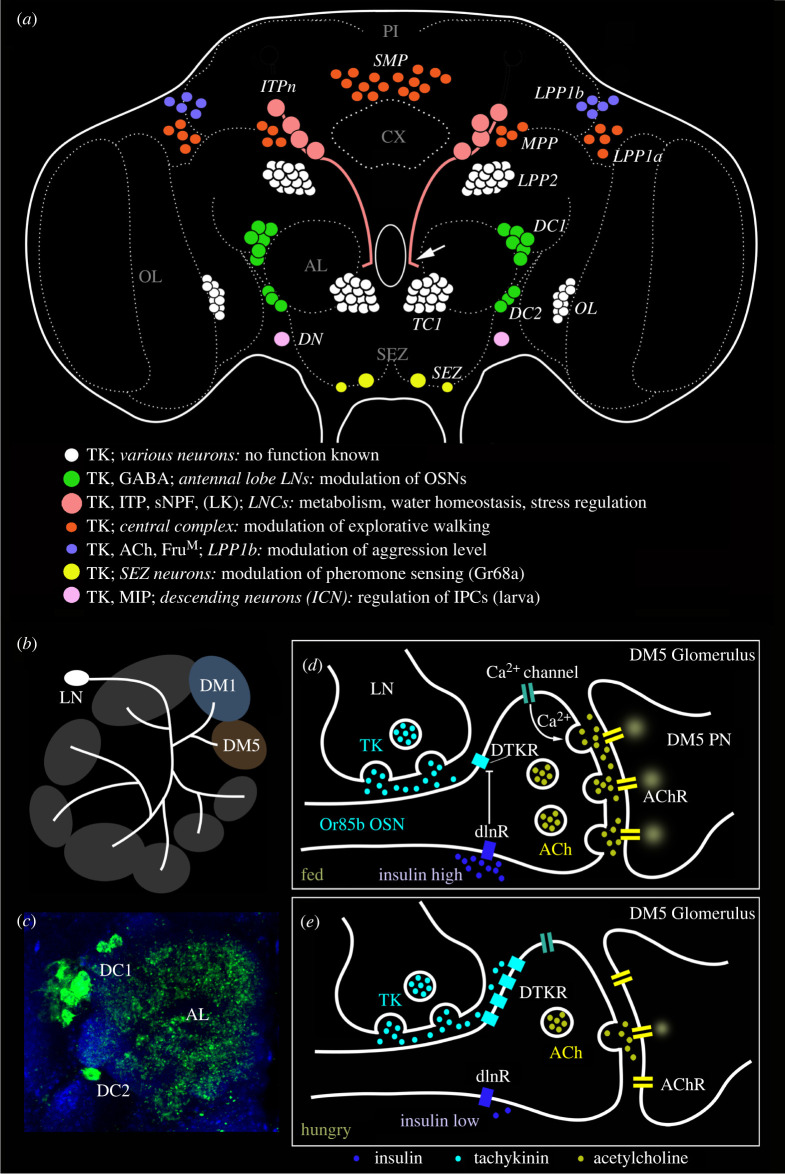
Executive peptide signalling: multiple distributed local functions illustrated by the numerous neurons producing tachykinin (TK). (*a*) Schematic of neuronal TK distribution in the adult *Drosophila* brain (frontal view). Neuronal cell bodies are shown in different colours (see legend in figure). Some have been studied functionally in some detail (several colours); others remain unexplored (white). The light red neurons (SMP, MPP, LPP1a) innervate different layers of the fan-shaped body of the central complex (CX) [97] and modulate explorative walking [98]. The green ones (DC1, DC2) are local neurons of the antennal lobe that are part of circuitry that modulates odour sensitivity in olfactory sensory neurons (OSNs) [99]. In male flies the light blue neurons (LPP1b) express FruM and probably acetylcholine (Ach) and regulate levels of aggression [100]. The pink ones (ITPn) are lateral neurosecretory cells that co-express TK, ion transport peptide (ITP) and short neuropeptide F (sNPF) [101] and regulate aspects of metabolic and water homeostasis [101,102]. The arrow indicates axons destined for peripheral neurohemal sites. The DN neurons are involved in pheromone sensing [103] and the SEZ neurons in regulation of larval insulin-producing cells (IPCs) [104]. The terminology (except ITPn) is from Winther *et al.* [105] and specifications of neurons are compiled from papers cited above. This figure was updated from Nässel *et al.* [106]. (*b–e*) Executive (intrinsic) peptidergic neuromodulation in the *Drosophila* antennal lobe, exemplified by TK signalling in modulation of food odour sensing. (*b*) TK peptides are expressed in local neurons (LN) of the antennal lobe and innervate most glomeruli. Two glomeruli are shown here (DM1 and DM5). Of these, DM1 mediates food odour attraction (Or42b) and DM5 food odour aversion (Or85b). (*c*) Image of TK immunoreactive LNs (green) in the clusters DC1 and DC2 innervating the antennal lobe (AL). (*d*,*e*) Role of TK signalling in the DM5 glomerulus, which relays aversive odour signals from olfactory sensory neurons (OSNs) that express odorant receptors Or85b to DM5 projection neurons (PN), which in turn signal to higher order neurons that control food search. (*d*) In fed flies the circulating level of insulin-like peptides (DILPs) is high, which suppresses expression of the TK receptor DTKR. When DTKR signalling is low there is no suppression of Ca^2+^ channel activity. Hence there is an increased release of acetylcholine (ACh) when the OSN is activated and as a consequence the DM5 PN relays strong aversive signals and food search is reduced. (*e*) In the hungry fly the DILP level is low, DTKR expression is high and therefore TK signalling activates DTKR and the OSN releases less Ach. This suppresses activation of the aversive DM5 PN and results in increased food search. The (*b*), (*d*) and (*e*) were redrawn from figures in Ko *et al.* [107].

Some specific neuropeptides are mostly expressed in numerous locally restricted neurons with small cell bodies, or even in sensory neurons, like olfactory sensory neurons (OSNs) (figures 2*b–d* and 3). Here the neuropeptide acts circuit-specifically and performs executive (intrinsic) modulation at specific synapses (e.g. potentiate or diminish, prolong or shorten a pre- or postsynaptic response). Peptides of this kind are in many cases known to be localized together with SMNs and thus likely to act as *co-transmitters* [18,36]. Examples of locally acting neuropeptides in *Drosophila* that have been fairly well studied are short neuropeptide F (sNPF), proctolin and tachykinin (TK) (see [18,36]). Each of these peptides appears to be multifunctional and their function is localized and dependent on their site of action in a circuit. Thus, these peptides are used for *executive neuromodulation.* Modulated systems include sensory cells where neuropeptides (e.g. sNPF and TK) mediate a gain control by setting sensitivities to, for example, odours, tastants and pain [99,107–110] (figures 2*b–d* and 3). Note that the executive peptide signalling can be influenced by upstream orchestrating signals that regulate peptide receptor expression (figure 2*d*) (see also §5.1). At the output side, the neuropeptide proctolin in motoneurons modulates glutamatergic signalling to muscles [111–114]. Moreover, several types of neuropeptides act locally, alone or together with SMNs, in interneurons including several types of clock neurons, intrinsic Kenyon cells of the mushroom bodies and in the central complex (see for example [36,97,115–119]).

In addition to the abovementioned neuronal systems, peptide hormones are also important for modulation of physiology and behaviour (figures 1*g* and 2*c*,*d*). They are used for interorgan communication and are produced by neurosecretory cells of the brain and ventral nerve cord (VNC) for signalling to the periphery (e.g. to renal tubules, fat body and intestine) or by EECs of the midgut for signalling to brain and other organs, commonly related to nutrition and metabolism [18,31,32,44,120–122]. Also, tissues like the fat body release peptide hormones or cytokines for interorgan communication related to metabolism and stress [32,122]. The signals from the EECs and fat body are important regulators of behaviours that are dependent on metabolic and nutritional status.

## 4. Neuromodulation in circuits supervising behaviours: endocrine cybernetics

Before turning into the mechanisms of peptidergic signalling in specific circuits that regulate behaviour, we shall briefly look into the organization of some key brain centres and their relations to peptidergic systems. To ensure appropriate state-dependent behaviours, there are hierarchically arranged regulatory brain circuits adjusted by multiple feedbacks (see e.g. [2,3,8,12,123–125]). Many of these synaptic circuits are immensely complex, like the central complex and the mushroom bodies (figure 1*a*), both of which interconnect to multiple areas of the brain [73,74]. In *Drosophila* the central complex has been shown to integrate numerous sensory modalities and brain regions and thereby coordinate activity and sleep, flexible context-dependent navigation and head direction, as well as visual learning relevant to orientation (see [73,126–128]). Thus, its circuitry controls activity and how the moving fly interacts with its surrounds. Also, in other insects the central complex is important in navigation and foraging (see [126,129,130]). Due to its unique organization as a fused neuropil in the brain midline and its wide connections to other brain regions, the central complex has been dubbed the ‘brain within the brain’ by Strausfeld [131] (see also [132]); recent research on its multiple functions certainly supports this designation.

The mushroom bodies are primarily known for their role in olfactory associative learning and memory [133–136]. However, they also receive visual, gustatory, temperature and humidity inputs [74]. Furthermore, mushroom bodies are under influence of the sleep–wake, metabolic and energy state of the fly (including hunger and thirst), and after integrating these signals they play roles in activity and sleep, control of context-dependent foraging and feeding, and coordination of motor behaviour [74,137–140].

Both the central complex and the mushroom bodies are subject to intrinsic and extrinsic neuromodulation by monoamines and neuropeptides, which confers plasticity to the hardwired circuitry [18,97,141–146]. One part of the peptidergic modulation is by neurons intrinsic to the each of the two neuropil regions, and the other part is by means of large extrinsic neurons that interconnect different brain regions (see [18,20,35,97,136]). However, the aminergic neuromodulation in these neuropils appears to be entirely by extrinsic neurons, at least in *Drosophila* (see [97,144,146,147]).

As mentioned, the central complex and the mushroom bodies interact extensively with other parts of the brain to guide behaviour. Sensory inputs from the external and internal environment (including the circadian clock and sleep homeostats) induce short- and long-term alterations of behaviour where neuromodulatory circuits are important. Of special interest in modulation of circuits in the mushroom body and central complex, and their associated brain centres, are large peptidergic interneurons that interconnect several portions of the fly brain. Many of these are uniquely identifiable and possible to target genetically, and thus their role in modulation of specific behaviours can be tested. This peptidergic signalling is to a large extent paracrine and mediating high-level endocrine modulation or even orchestration. Thus, in the following we will deal mostly with a non-synaptic domain of the CNS and with context- or state-dependent neuromodulation that supervises behaviour and coordinates physiology. Thus it is appropriate to talk about *endocrine cybernetics*, the regulation or governance of behaviour by paracrine chemical signalling. This is formulated by using a wider interpretation of Norbert Wiener's term *cybernetics* [148] or *biological cybernetics* (see also [149]) and considering the generic name *cybernin* given by Roger Guillemin to substances (peptides) that are used for paracrine signalling [63]. Thus, endocrine cybernetics describes the role of neuropeptides and peptide hormones in signalling that imparts high-level plasticity to neuronal networks that control behaviour (and associated physiology), thereby enabling animals to make state- and context-dependent behavioural decisions, including choice between conflicting behaviours.

Next, we shall look into specific examples of hierarchical organization of peptidergic neuromodulatory systems that integrate external stimuli and internal states to modulate behaviour and physiology, and how specific sets of neurons act as molecular switches between competing behaviours.

## 5. Examples of peptidergic regulation of state-dependent behavioural choices at different hierarchical levels

This section highlights how the internal state and external sensory cues influence peptidergic systems that in turn regulate behaviour and physiology. Modulation of behaviourally relevant circuits starts already at the first synapses in sensory systems with gain control mechanisms, continues in higher brain centres and finally affects the motor output to muscles. As mentioned, the modulatory peptidergic systems act at different hierarchical levels in the brain, from state/context-dependent orchestrating signalling by higher order neurons, to the lowest level with local executive modulation in specific circuits. We start at the executive level and continue up the levels.

### 5.1. Circuit-specific executive neuropeptide signalling

Neuropeptides that act locally in executive signalling are commonly widely expressed in many neurons (160 to more than 1000) in multiple locations of the *Drosophila* brain. Yet, the peptide signalling can be influenced by upstream signals (including hormonal ones) that regulate peptide or G protein-coupled receptor (GPCR) expression, for example. One example of a widely distributed neuropeptide with *multiple local functions* in *Drosophila* is TK shown in figure 3 [106]. sNPF is another peptide that acts at short range with multiple localized executive functions (see [150,151]). It is produced by several hundred neurons in the CNS, as well as in more than thousand Kenyon cells of the mushroom bodies [150]. The neurons expressing sNPF additionally include subsets of chemosensory cells of the antennae and maxillary palps, two sets of lateral neurosecretory cells (LNCs) and many types of small interneurons in the visual system, central complex, clock system and other brain regions [101,116,150]. Next, we describe executive neuromodulation by TK and sNPF.

The cell bodies of the numerous TK producing neurons are shown in figure 3*a*. Clusters of TK expressing neurons innervate distinct neuropils of the brain such as central complex, antennal lobes and optic lobes, but also neuropils interspersed between these and the pars intercerebralis where the median neurosecretory cells reside. Different functions of TK neurons are shown in figure 3*a*. It can be mentioned that there are two GPCRs that respond to TKs, TkR86c and TkR99D [152,153]. One of these, TkR99D, is a bona fide TK receptor [154], the other (TkR86c) has been shown to respond to one TK isoform (TK6) as well as to isoforms of the peptide natalisin [155,156]. Interestingly, as we shall see below, some modulatory functions of TKs in the brain seem to be mediated by TkR99D and others by TkR86c. Thus, TK signalling operates in a differential and divergent fashion and seems to overlap partly with natalisin signalling.

The TK neurons that innervate different layers of the fan-shaped body of the central complex modulate neurons that control explorative walking [97,98]. In the antennal lobe, local TK neurons, some of which co-express GABA, are part of circuitry that regulates odour sensitivity in olfactory sensory neurons (OSNs) by action on TkR99D [99,107]. This modulation will be described in more detail below. In male flies, one set of TK neurons (LPP1b) express the male splice form of the transcription factor fruitless (Fru^M^), and probably acetylcholine (Ach), and regulate levels of aggression via TkR86C [100]. A small group of TK neurons act on TkR86C, expressed by specific clock neurons, to modulate daily locomotor activity [157]. Other subsets of the TK neurons modulate pheromone sensing and regulate activity in the insulin-producing cells (IPCs) [103,104,158,159]. The TK regulation of IPCs is by means of TkR99D [158,159]. Finally, TK is expressed in a set of LNCs designated ITPn or ALK that have axon terminations in the corpora cardiaca–corpora allata (CC–CA), anterior aorta and foregut [101]. These cells co-express TK, ion transport peptide (ITP) and sNPF (and possibly LK) and regulate aspects of metabolic and water homeostasis [101,102,160]. A specific role of TK in these neurons was seen in regulation of responses to metabolic and desiccation stress [101]. There is no evidence that TK and sNPF in these ITPn/ALK neurons are released as circulating hormones. Possibly, they act as local modulators of hormone release (ITP release by autocrine regulation, or other hormones released from adjacent axon terminations in CC–CA). They may additionally be released in a paracrine way within circuits of the brain. Additionally, it has been shown that TK in intestinal EECs regulates lipid metabolism, DILP3 signalling and stem cell homeostasis locally in the midgut [161–163]. In summary, the above examples suggest that TK functions in different circuits as a local executive neuromodulator, and at present there is no evidence for any coordinated action of TK in the different circuits.

To illustrate executive modulation at specific synapses, we next focus on the TK action as a neuromodulator in the olfactory system. A number of local interneurons (LNs) of the antennal lobe express TK and GABA, and subsets of the OSNs express the TK receptor TkR99D [99] (figure 3*b–e*). It was shown that TK signalling from LNs to OSNs mediates a presynaptic inhibitory feedback by suppressing calcium and synaptic activity in OSNs [99]. Next, a modulatory role of TK signalling in sensing food odours at this first synapse was unveiled [107]. In fed flies, where circulating levels of insulin-like peptides (DILPs) are high, the TK receptor is downregulated in OSNs carrying specific odorant receptors (Or42b and Or85a) (figure 3*d*). In the antennal glomerulus DM5, which conveys food odour aversion (negative valence), downregulation of the inhibitory TK receptor in a fed fly leads to decreased (inhibitory) presynaptic TK signalling. This results in increased synaptic release of acetylcholine and as a consequence increased activation of the antennal lobe projection neurons (PNs) with aversive valence leading to increased food aversion [107]. In hungry flies, where circulating levels of DILPs are low, the TK receptor is upregulated in the OSNs (figure 3*e*). Upregulation of the inhibitory TK receptor leads to increased presynaptic TK signalling and thus suppressed depolarization. Consequently there is a decreased synaptic activation of antennal lobe PNs leading to increased food attraction [107].

In addition to TK, there is a parallel system that uses sNPF for presynaptic modulation of odour signalling in specific olfactory glomeruli [108] (electronic supplementary material, figure S1). In the DM1 glomerulus (positive valence; wired for food odour attraction), which is innervated by Or42b- and sNPF-expressing OSNs, enhanced signalling with sNPF increases food attraction in hungry flies with low circulating DILPs [107,108]. This enhanced signalling is caused by upregulation of sNPF receptor expression on OSNs and strengthened synaptic activation of PNs by increased acetylcholine release from OSNs (electronic supplementary material, figure S1). This local executive peptide signalling is thus mediated presynaptically (autocrine signalling) by subsets of OSNs. Both the TK and the sNPF signalling are under regulation by nutrient-dependent DILP signalling. Together, peptidergic neuromodulation of the two odour channels (DM1 and DM5) ensures that hungry flies increase food search. This state-dependent modulation of executive peptide signalling is also illustrated in figure 2*d*. That figure additionally shows the influence of state-dependent signalling from the large SIFa neurons on food attraction [87], illustrating how global neuronal and hormonal signalling act together to modulate local peptidergic signalling at peripheral synapses in the olfactory system in a state-dependent fashion.

Other executive roles of sNPF can be seen in different circuits of the brain. This peptide acts as a presynaptic neuromodulator of acetylcholine signalling in intrinsic neurons of the mushroom body [115,164], as a co-neuromodulator with pigment-dispersing factor (PDF) in small lateral clock neurons (sLNvs) in a circuit mediating phase-setting (light entrainment) [165], and in interneurons as a neuromodulator of explorative walking in circuits of the central complex [98]. Other local roles of sNPF include regulation of sleep [166,167], food intake [20,168], modulation of gustatory receptors [169] and nociceptive circuits [170]. These neuromodulatory actions appear to be regionalized and functionally independent. However, it is possible that some of them are targets of global modulation, similar to the sNPF action in the olfactory system (electronic supplementary material, figure S1), since they are associated with appetitive behaviour and therefore nutrient dependent.

### 5.2. Context-specific signalling by intermediate size peptidergic interneurons that integrate various modalities

Many neuropeptides in the *Drosophila* brain are produced in anatomically mixed populations of interneurons, both with local and more wide-field branching, in numbers ranging between about 10 and 100. Some of the same peptides are also expressed by neurosecretory cells and EECs. The majority of the different neuropeptides present in *Drosophila* interneurons probably fall into this category. Here, we use LK- and AstA-producing neurons as examples to illustrate context-specific neuromodulation by neurons that receive inputs from different modalities and interconnect various circuits to confer context-specific plasticity to behaviour and associated physiology.

#### 5.2.1. Leucokinin signalling

**Figure 4.**
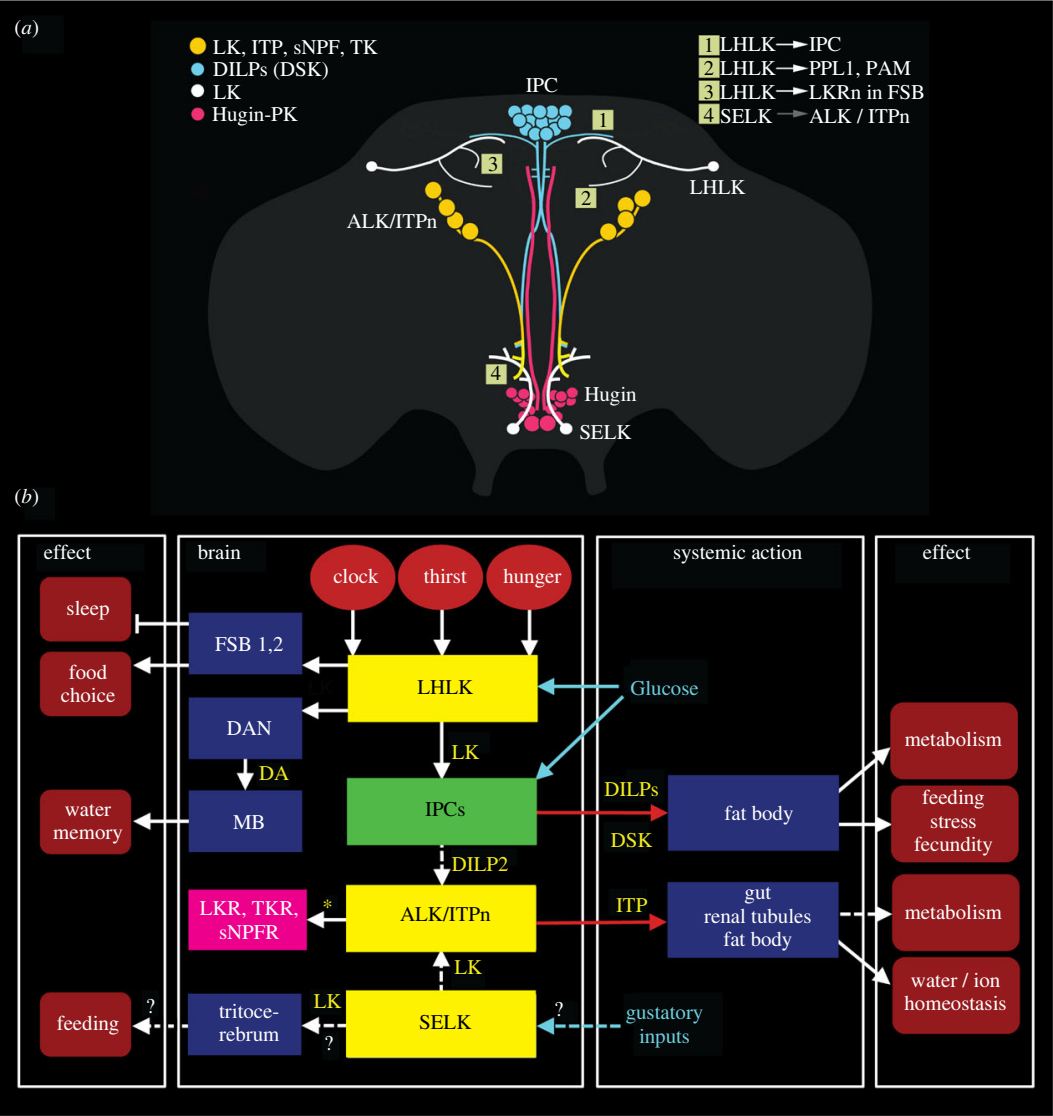
Context-specific signalling, exemplified by neurons signalling with leucokinin (LK). (*a*) Schematic depiction of LK neurons in relation to some neurosecretory cells in the adult *Drosophila* brain. The LHLKs act on (white arrows) insulin (DILP) producing cells (IPCs), dopaminergic neurons (PPL1 and PAM subtypes) and at least two types of LK receptor (LKR)-expressing neurons (LKRn; FSB 1 and 2; see (*b*)) innervating the FSB. SELKs may act (grey arrow) on the ALK/ITPn that express the LKR. The Hugin neurons of the suboesophageal zone are shown since they form a link between gustatory sensory cells and feeding circuits, including IPCs. The numbered boxes (1–4) indicate sites of interaction between neurons. Data derived from [9,93,94,172,173]. (*b*) Schematic diagram of functional connections between LK neurons (yellow boxes) and other neurons, circuits and peripheral targets. Arrows indicate various actions, dashed arrows (and ?) suggest actions yet to be confirmed, and stop bars indicate inhibitory action. The LK neurons in the brain are shown as yellow boxes and the IPCs as a green box. Targets of LK signalling are shown as dark blue boxes. LHLKs signal to two types of LKR expressing neurons of the fan-shaped body (FSB 1, 2; these are LKR neurons and FBl6 neurons), and via dopaminergic neurons (DAN) to mushroom body-associated neurons (MB). The LKR-expressing FSB neurons inhibit sleep [172] and the FBl6 neurons regulate food choice [9], whereas the MB neurons, via dopamine (DA) inputs, mediate water (and sugar) memory [34]. LHLKs respond to decreased glucose and receive inputs from neuronal circuits of the circadian clock and systems sensing thirst and hunger (red ellipses). LHLKs signal with LK to IPCs, which regulates sleep–metabolism interactions [93,94]. IPCs are nutrient-sensing and use DILPs to regulate multiple functions, including carbohydrate and lipid metabolism, feeding, stress responses and fecundity; IPCs also express drosulfakinin, DSK (see [174]). IPCs are likely to act on the ALK/ITPn with DILP2 (dashed arrow) (see [175]). SELK neurons may receive gustatory inputs [171], but their actions are not functionally confirmed (dashed arrows and ?). The ALK/ITPn are neurosecretory cells that use ion transport peptide (ITP) to systemically regulate water homeostasis via the intestine and hindgut and also to regulate feeding and drinking [102]. These cells also use tachykinin (TK) and short neuropeptide F (sNPF) to regulate responses to starvation and desiccation [101], probably by paracrine signalling (asterisk), but the neuronal circuitry is not yet known. The magenta box represents neurons expressing receptors for LK, TK and sNPF (and ITP; not shown) in the brain that are yet to be identified. The role of LK in ALK/ITPn cells is not yet known. (*b*) is updated and modified from Nässel [176].

LK is consistently expressed by two pairs of neurons in the *Drosophila* brain, one pair of LHLKs and one of SELKs [171] (figure 4*a*). In addition, eight larger neurosecretory cells (ALKs or ITPn) display LK immunoreactivity and *Lk*-Gal4 expression in early larvae, but only the latter in adult flies; they also produce ITP, sNPF and TK [94,101,171] (figure 4*a*). In the adult abdominal ganglia, a set of 20–22 LK-producing neurosecretory cells, ABLKs, co-express diuretic hormone 44 (DH44) and are involved in regulating water and ion homeostasis [96]. The multiple inputs and outputs of LK neurons in the brain are shown in figure 4*b*.

**Figure 5.**
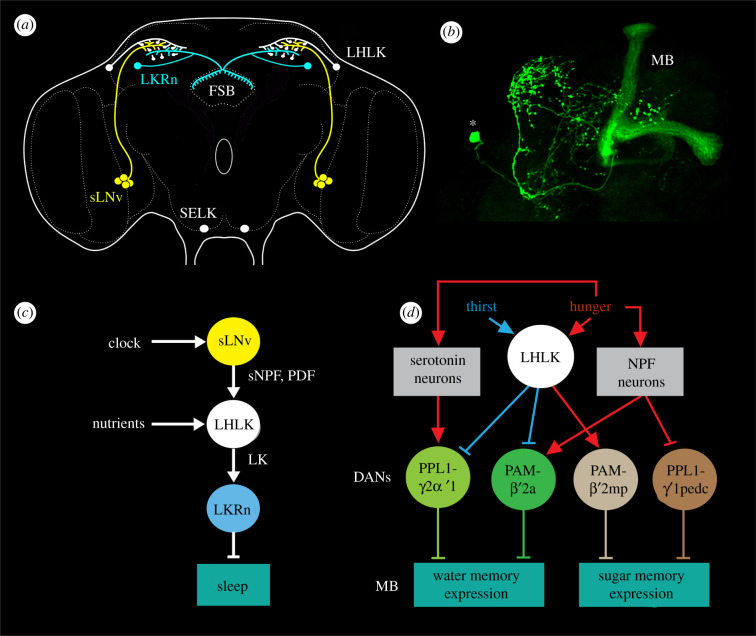
Context-specific LK signalling in regulation of sleep–metabolism interactions and water memory in *Drosophila*. (*a*) Schematic of brain neurons connecting clock, nutrient-sensing and sleep regulation. There is one pair of LHLK neurons in the brain. Clock neurons (sLNv) have outputs on each LHLK that in turn inhibit LK receptor expressing neurons (LKRn) that innervate the fan-shaped body (FSB) and thereby inhibit sleep in a nutrient-dependent fashion. Figure compiled from data in [93,172,178,179]. (*b*) Image of the LHLK neuron in the left hemisphere of the brain, with cell body at *. MB, mushroom body. From Zandawala *et al.* [94]. (*c*) A schematic of the connections in (*a*). PDF, pigment-dispersing factor; sNPF, short neuropeptide F. (*d*) LHLK neurons and a circuit regulating water–sugar based memory. The LHLK neurons receive hunger and thirst signals and act on dopaminergic neurons (DANs) of PPL1 and PAM subtypes to regulate expression of water and sugar memory in mushroom body (MB) circuits. Some of these neurons also receive inputs from serotonin and neuropeptide F (NPF) producing neurons. Redrawn from Senapati *et al.* [34].

Of the four consistently LK-expressing brain neurons only the pair of LHLKs has been specifically investigated. These LK neurons co-express glutamate [177], the RNA/DNA binding protein translin [178] and 5′ adenosine monophosphate-activated protein kinase (AMPK) [93] and modulate several brain circuits that regulate feeding, sleep–metabolism interactions, and state-dependent memory formation (see figures 4*b* and 5; electronic supplementary material, figure S2). Each of the two LHLKs is restricted to the dorsolateral region of one brain hemisphere (figure 5*a*,*b*), receives inputs from clock neurons (sLNv) and connects to the IPCs, dopaminergic neurons (DANs) innervating the mushroom bodies and LK receptor (LKR) expressing interneurons that innervate the fan-shaped body of the central complex (figure 4*a*) [9,34,93,94,172,178,179]. The activity in LHLKs is dependent on the fly's glucose levels [93] and water homeostasis [34] and form an interface between the circadian clock, nutrient status (thirst and hunger) and different circuits that regulate sleep, water and sugar memory expression and food choice [9,34,93,94,172,178,179] (figures 4*b* and 5*c*,*d*; electronic supplementary material, figure S2). The involvement of LK signalling in food choice has not been confirmed as specifically mediated by the LHLKs, but is dependent on nutrient inputs to the LK neurons [9] (electronic supplementary material, figure S2). Interestingly, the LK-regulated food choice behaviour relies on neurons in the fan-shaped body (FBl6 neurons) that integrate multiple state- and experience-dependent inputs from peptidergic neurons and hence modulate feeding motor circuits. The FBl6 neurons are involved in making a final experience-based food choice when flies are exposed to conflicting gustatory stimuli and they also receive inputs from AstA and DH44 producing neurons [9]. Thus, LK signalling to circuits in the fan-shaped body (FSB) regulates both food choice and nutrient-dependent sleep [9,172,179]. A further role of the LHLKs in metabolic regulation of sleep is mediated by action on the IPCs [93]. The IPCs express the LKR and it was shown that this receptor is required for starvation-dependent sleep suppression and that LHLKs mediate metabolic state to the IPCs [93]. Thus, the LHLKs relay nutritional state and circadian timing to several circuits that regulate sleep, food choice, water and sugar memory and feeding. These circuits reside in the fan-shaped body, the mushroom bodies and the pars intercerebralis that houses the IPCs. In summary, the single pair of glucose-inactivated LHLKs serves as a good example of neurons that mediate state/context-mediated signals, not globally, but to several circuits that regulate different behaviours.

#### 5.2.2. Allatostatin-A signalling

**Figure 6.**
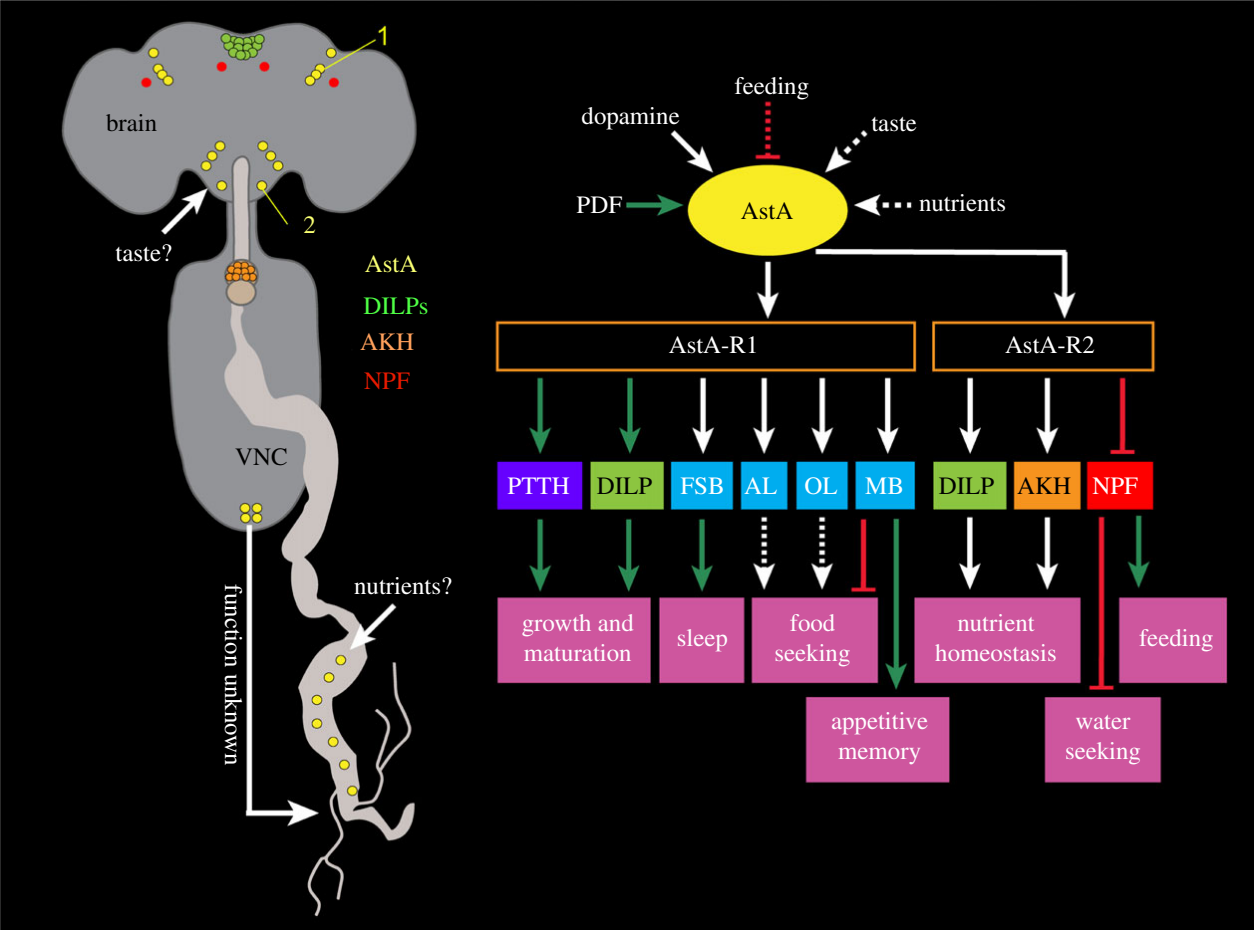
Context-specific signalling with Allatostatin-A (AstA). AstA neuropeptide from the brain and gut regulates diverse feeding associated behaviours and physiology. (*a*) A schematic showing the locations of select AstA-expressing cells in the nervous system and midgut, as well as some of its downstream neuronal targets. The morphology and location of AstA cells suggest that the suboesophageal zone AstA neurons may receive taste inputs from the proboscis, and AstA-expressing enteroendocrine cells in the gut may be nutrient-sensitive. A PLP neuron is indicated by 1 and a Janu-AstA by a 2; these are discussed in the text. (*b*) Inputs and behavioural outputs of AstA cells. AstA-expressing neurons receive inputs from the pigment-dispersing factor (PDF) expressing clock neurons [91] and dopaminergic inputs via the Dop1R1 receptor [9]. In addition, feeding inhibits AstA neurons and they may also receive gustatory inputs via the proboscis and nutrient information via the gut. AstA, in turn, mediates its effects via its two receptors, AstA-R1 and AstA-R2 expressed in various peptidergic cells/neurons. These include cells expressing prothoracicotropic hormone (PTTH), insulin-like peptides (DILPs), adipokinetic hormone (AKH) and neuropeptide F (NPF). AstA-R1 is also expressed in neurons in the fan-shaped body (FSB) and mushroom body (MB). Moreover, axonal projections of AstA neurons to the antennal lobe (AL) and optic lobe (OL), coupled with single-cell transcriptome data from the Fly Cell Atlas suggest expression of AstA-R1 in the AL and OL. AstA modulation of peptidergic neurons and other neuronal targets influences various feeding-related behaviours including feeding, appetitive memory and water seeking. Green arrows represent stimulation, red bars represent inhibition, white arrows represent unclear valence and dashed arrows represent postulated actions. (*b*) is based on [9,20,91,92,180–183].

In *Drosophila* there are about 20 AstA interneurons in the midbrain, and a large number in the medulla of the optic lobes [95] (figure 6*a*). In the VNC, there are approximately 14 pairs of AstA neurons, three of which innervate the hindgut; four neuroendocrine cells are associated with dorsolateral VNC nerve roots [95]. Furthermore, AstA peptides are produced by EECs in the midgut [40,95] (figure 6*a*). In adult flies, AstA acts as a neuromodulator in regulation of food and water search, sugar reward, feeding, metabolism and sleep (figure 6*b*) [20,91,92,180–182,184,185]. The hormonal role of gut EEC-derived AstA is not yet clear (see [91]) (figure 6*a*). AstA mediates its actions via its two receptors related to mammalian galanin receptors, AstA-R1 and AstA-R2 expressed in various peptidergic cells/neurons. The functional division of these receptors is shown in figure 6*b*.

Two sets of AstA-expressing brain neurons have been specifically investigated, the PLP neurons in dorsal protocerebrum and Janu-AstA neurons in the SEZ [91,92] (figure 6*a*). Both have wide arborizations interconnecting several brain regions: the three pairs of PLPs arborize in neuropils in the entire dorsal protocerebrum and the pair of Janu-AstA neurons arborize in the suboesophageal zone (SEZ) and send ipsilateral axons dorsally that branch in the superior median protocerebrum [91,92].

The PLP neurons receive inputs from the PDF and sNPF-expressing sLNv clock neurons and release AstA to target several types of neurons in the posterior superior protocerebrum that regulate sleep, as well as interact with IPCs and probably adipokinetic hormone (AKH) producing cells to mediate satiety and food choice [91,180,184] (figure 6*b*). It was suggested that also AstA-expressing EECs may partake in this signalling and additionally regulate gut peristalsis [91]. These authors proposed that the clock-controlled PLP neurons together with EECs signal to ensure an energy-saving sleep state with lowered digestive activity and metabolism [91]. Probably the PLP neurons are the ones signalling to AstA-R1-expressing sleep promoting neurons in the dorsal layer of the fan-shaped body and thereby promote rest [184].

The Janu-AstA neurons in the SEZ were shown to promote water seeking and inhibit feeding by activating AstA-R2 receptors on NPF neurons [92]. These neurons do not regulate water intake, only search for water in thirsty flies. Thus the Janu-AstA neurons and a subset of NPF neurons (that respond to thirst) reciprocally regulate behaviours related to hunger and thirst (figure 6*b*).

Global interference with AstA signalling has revealed further AstA action where the specific peptidergic neurons remain unidentified. Since there are only a few types of AstA-expressing neurons in the fly brain and most are rather widely arborizing, we speculate that these experiments interfered with intermediate sized neurons that mediate state/context-mediated signals (mode 2). A satiety-inducing role of AstA neurons was shown where activation of these neurons caused food aversion and decreased motivation to feed [181]. Furthermore, AstA mediates satiation via DANs that modulate mushroom body output neurons (MBONs) [20]. This inhibitory pathway suppresses food-seeking behaviour (figure 6*b*). Another circuit involving DANs that act on mushroom body-associated neurons mediates sugar reward in appetitive memory formation and is inhibited by satiety-inducing AstA [182] (figure 6*b*). Furthermore, AstA acts on AstA-R1-expressing neurons in layer 6 of the fan-shaped body to modulate taste-dependent food choice [9] (figure 6*b*; electronic supplementary material, figure S2). Thus, in summary, AstA neurons translate a state of satiety into behavioural changes that result in rest/sleep and reduced attraction to food. The AstA neurons monitor internal states and external stimuli by inputs from the circadian clock, nutritional state, gustatory inputs and are modulated by dopaminergic inputs (figure 6*b*). As seen in figure 6*b*, the AstA neurons also play regulatory roles in growth and maturation during larval development [183]. A recent review summarizes pleiotropic AstA functions also in other invertebrates [185].

### 5.3. Orchestrating signalling by peptidergic interneurons

A few *Drosophila* neuropeptides are produced by a small number of extensively arborizing interneurons that mediate high-level neuromodulation that affects behavioural choices. We shall look into two such systems that have been investigated in some detail, neurons expressing SIFa and DSK.

#### 5.3.1. Orchestrating signalling by SIFamide neurons in the brain

**Figure 7.**
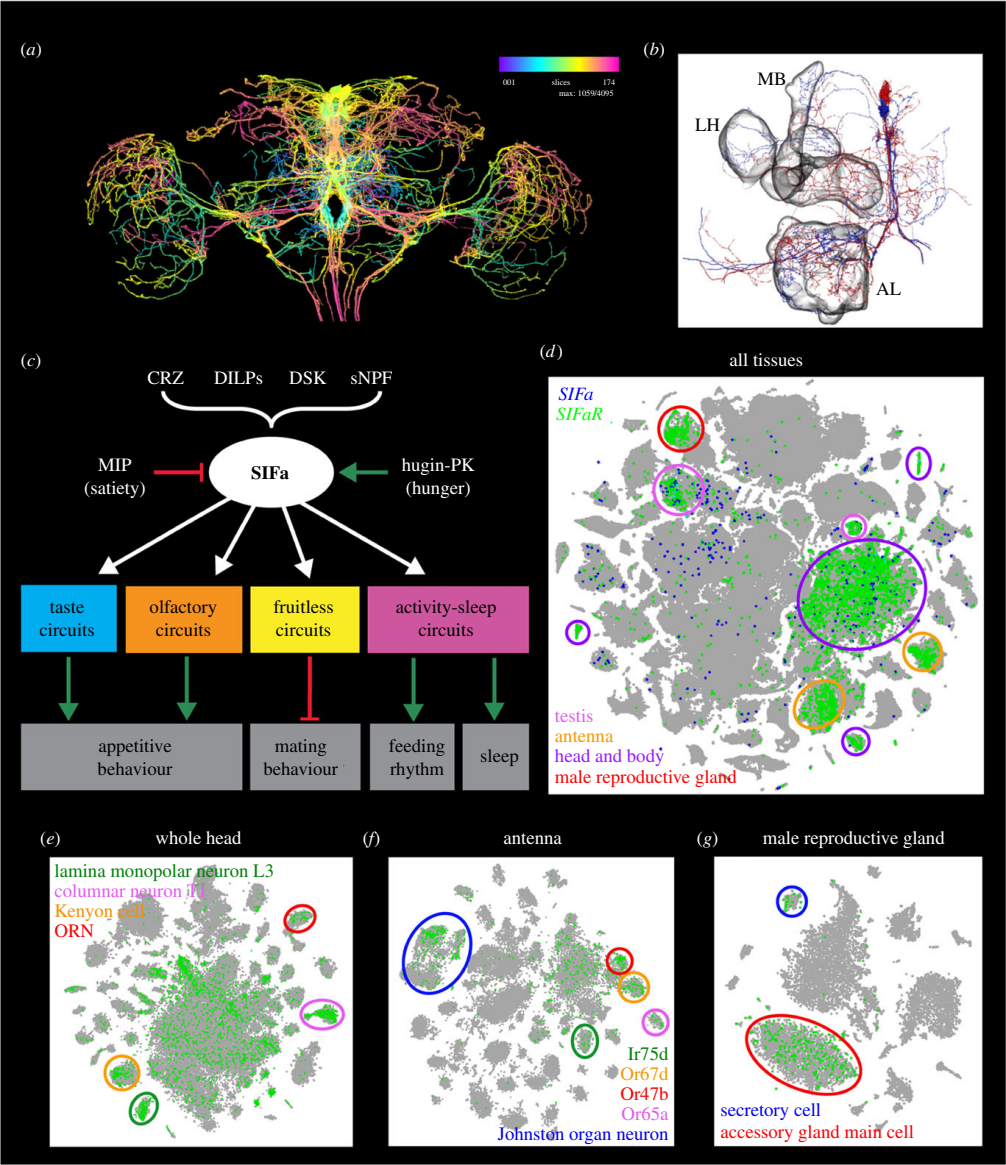
Orchestrating neuromodulation and behavioural switches illustrated by SIFamide (SIFa) neurons. Expression of SIFa and SIFaR correlates with the functions of this signalling system. (*a*) SIFa expression in four neurons with extensive arborizations throughout the brain, including the central complex, mushroom bodies antennal and optic lobes. Image obtained from https://neuronbridge.janelia.org [188,189]. (*b*) Partial reconstructions of two SIFa neurons from serial electron microscopic sections. MB, mushroom body, LH, lateral horn, AL, antennal lobe. Data obtained from neuPRINT (https://neuprint.janelia.org) [188,190]. (*c*) Peptidergic inputs and behavioural outputs of SIFa neurons. SIFa influences appetitive behaviour via modulation of taste (neurons not identified) and olfactory circuits (neurons in DM3 glomerulus; see also (*f*)), mating via actions on fruitless-expressing neurons and sleep via modulation of activity-sleep circuits. CRZ, corazonin; DILPs, insulin-like peptides; DSK, drosulfakinin; sNPF, short neuropeptide F; MIP myoinhibitory peptide, Hugin-PK, Hugin pyrokinin. Based on [87,90,186,191,192]. (*d*) t-SNE visualization of single-cell transcriptomes derived from all the tissues of the adult fly. SIFa and SIFaR are expressed in the head and body, and testis clusters whereas SIFaR is additionally expressed in the antenna and male reproductive gland. (*e–g*) t-SNE visualization of single-cell transcriptomes showing the expression of SIFaR in different cell populations of the (*e*) whole head, (*f*) antenna and (*g*) male reproductive gland. Within the whole head, SIFaR is expressed in the lamina monopolar neurons L3, columnar neurons T1, Kenyon cells of the mushroom body and olfactory receptor neurons (ORNs). SIFaR is broadly expressed in the antenna with prominent expression in the Johnston organ and olfactory neurons expressing Ir75d, Or67d, Or47b and Or65a odorant receptors. Lastly, SIFaR is predominantly expressed in the male accessory gland main cells and secretory cells of the male reproductive gland. Data for (*d–g*) were mined using Scope (http://scope.aertslab.org) [193].

Four neurons with cell bodies in pars intercerebralis and extensive branches throughout the *Drosophila* brain and VNC express SIFa [186,187] (figure 7*a*,*b*). They are probably the most widely arborizing peptidergic neurons in *Drosophila*. The SIFa neurons have been shown to integrate several inputs and form a hub that regulates several behaviours, some of which are conflicting (figure 7*c*) as will be discussed in some detail. Interestingly, it seems that SIFa transcript is produced also in the testis (FlyCellAtlas; https://www.flycellatlas.org/) (figure 7*d*), but no function of this is known. Not surprisingly, the SIFa receptor (SIFaR) has a widespread distribution in many neuron types and other cells (figure 7*d–g*).

The SIFa neurons receive inputs from peptidergic neurons that mediate hunger (Hugin-pyrokinin) and satiety (myoinhibitory peptide) signals [87] (figure 7*a*). They are likely to also receive inputs from other peptidergic pathways, based on anatomical findings [87,194]. Some of these are indicated in figure 7*a*. The SIFa neurons interact with male-specific Fru^M^ neurons and inhibit male mating behaviour and regulate experience-dependent mating duration [186,191,195]. The mating duration is modulated via SIFamide receptor expressing neurons that signal with AKH, AstA, LK, crustacean cardioactive peptide (CCAP), FMRFamide and myosuppressin [195]. Furthermore the SIFa neurons target olfactory and gustatory neurons to stimulate appetitive behaviour and increase acute feeding behaviour [87]. Single-cell transcriptomics indicates expression of the SIFaR in olfactory sensory neurons and mushroom body neurons (figure 7*e*,*f*), both of which play roles in appetitive behaviour.

Another study showed that the SIFa neurons and SIFa peptide are critical for the timing of feeding and regulate the strength of the feeding rhythm [90]. Moreover, the SIFa neurons contribute to circadian locomotor activity and acutely promote sleep [90,192,196] (figure 7*c*). The effects of SIFa neuron activation on sleep is more prominent in females and the effect on mating larger in males, indicating sex-specific roles of these neurons and SIFamide [186,197]. Taken together data thus show that the four SIFa neurons weigh multiple sensory inputs, including clock signals, to regulate competing behaviours by stimulating food search, feeding, feeding rhythm and sleep, and inhibiting mating (figure 7*c*).

The complex anatomy of the SIFa neurons [186] suggest further regulatory roles, including possible feedbacks to clock neurons as indicated by transsynaptic trans-tango labelling [90]. In addition, connectomics analysis of the early larval brain has revealed complex input–output relations of SIFa neurons and other neurons in the pars intercerebralis [194]. Thus, it can be expected that the SIFa neurons will be found to be central in context-dependent orchestration of further behaviours both in larvae and adult flies.

#### 5.3.2. Orchestration and behavioural choice mediated by drosulfakinin-expressing neurons in the brain

**Figure 8.**
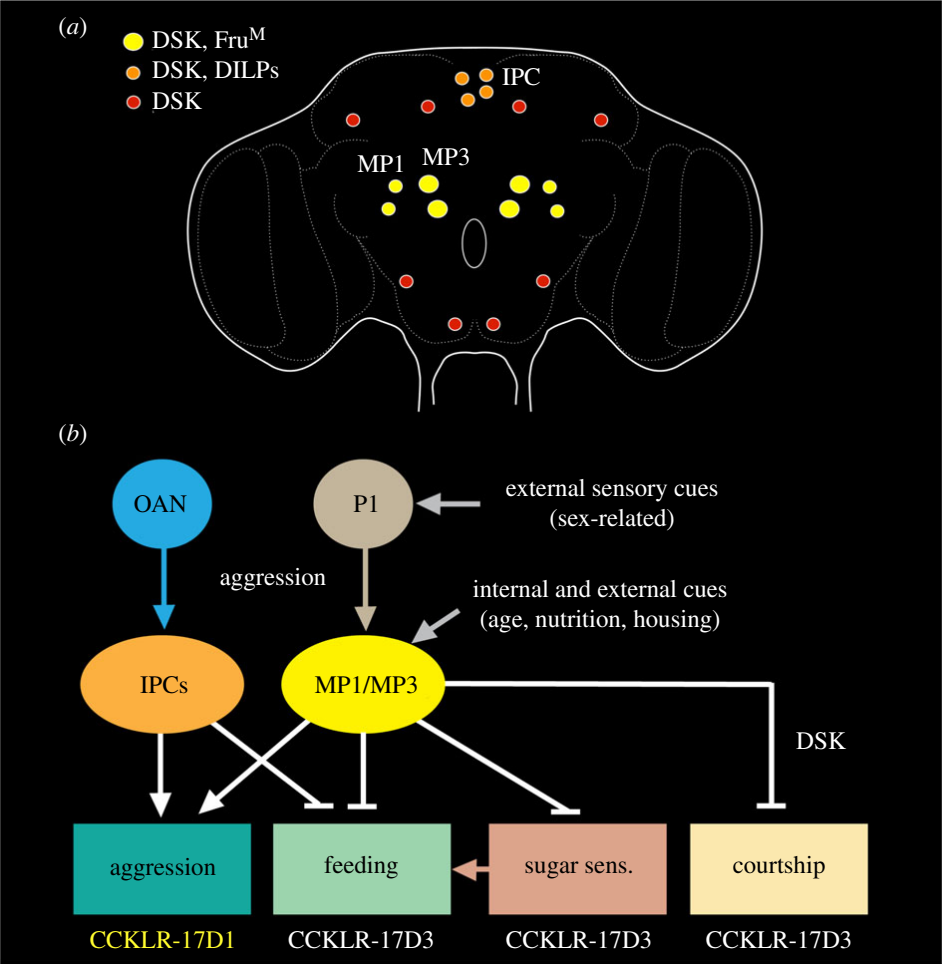
Orchestrating neuromodulation and behavioural switches illustrated by drosulfakinin (DSK)-producing neurons. (*a*) Distribution of DSK-expressing neurons. The neurons whose functions have been investigated are the MP1 and MP3 neurons that in male flies co-express fruitless (Fru^M^) and a subpopulation of the 14 insulin-producing cells (IPCs). (*b*) Regulatory roles of the DSK neurons in male flies. DSK signalling is shown with white arrows (activation) and stop bars (inhibition). Note that the MP1/MP3 neurons stimulate aggression (via the DSK receptor CCKLR-17D1) and inhibit courtship as well as sugar sensing gustatory receptors (Gr64f) and feeding (via CCKLR-17D3). DSK from IPCs stimulate aggression and inhibits feeding (but DSK receptor type was not investigated). The MP1/3 neurons are modulated by internal states and external cues as well as by a population of P1 neurons that mediate male-specific behaviours and receive sensory inputs from conspecific male and female flies (sex pheromones, visual, etc.). MP1/MP3 neurons also feed back onto P1 neurons to suppress courtship (not shown here, but see electronic supplementary material, figure S3). The IPC mediated aggression (and courtship; not shown) is regulated by OAN. For further details see electronic supplementary material, figure S3. This figure is compiled from [88,89,199–202].

About 20 neurons in the *Drosophila* brain express DSK, a peptide ancestrally related to cholecystokinin (CCK) [88,89,198] (figure 8*a*). Of these, two pairs of DSK neurons (MP1a and MP1b) have extensive bilateral arborizations within the brain and axons descending into the VNC [88,89] (figure 1*f*). These neurons are considered here in orchestration of behaviour together with the less elaborate ipsilateral MP3 neurons. Additionally, at least four of the 14 IPCs co-express DSK and DILPs [199] and will also be discussed here. To further increase signalling complexity, DSK uses two functionally distinct receptors, CCKLR-17D1 and CCKLR-17D3 [88,89,203,204].

Recent studies of male flies have implicated the MP1/MP3 neurons and the two DSK receptors in regulation of aggression, courtship behaviour, daytime locomotor activity, gustatory reception and feeding [88,89,200]. Thus, these neurons act in regulation of competing behaviours. First, we describe the role of DSK neurons in satiety and feeding.

An early report suggested that DSK acts as a mediator of satiety in *Drosophila* [199]. A later study confirmed this and additionally showed that food intake increases *Dsk* mRNa in fly heads and DSK immunolabelling in MP1/MP3 cells, as well as spontaneous electric activity and Ca^2+^ activity in MP1 neurons [200]. Furthermore, optogenetic activation of DSK neurons decreases the proboscis extension response and *Dsk* mutant flies displayed increased motivation to feed. Thus, feeding induces activation of MP1/MP3 neurons leading to DSK release that inhibits feeding behaviour. One action of DSK is to regulate the sensory gain of gustatory receptor neurons (GRNs) in the mouthparts and forelegs. The sugar receptor Gr64f in GRNs is downregulated in fed flies and upregulated in starved ones [200]. Knockdown of *Dsk* in MP1/MP3 neurons (but not IPCs) leads to an upregulation of Gr64f expression and downregulation of the gene *takeout*. Takeout is known to play an important role in the circadian activity and feeding behaviour [205,206]. Furthermore, Gou *et al*. [200] showed that *takeout* knockdown upregulates *Gr64f* expression. Activation of the DSK neurons decreases the sugar sensitivity of GRNs and taken together data suggest that feeding triggers release of DSK, which promotes *takeout* expression and downregulates Gr64f sugar receptor expression and thereby a reduction in activity in sugar-sensing GRNs [200] (electronic supplementary material, figure S3A,B). The same study showed that the DSK receptor CCKLR-17D3 is present in GRNs in the proboscis and proleg tarsi that co-express Gr64f. The expression of this DSK receptor is downregulated by feeding, and specific knockdown of CCKLR-17D3 in.Gr64f-expressing GRNs display a reduced motivation to feed. Knockdown of the other receptor CCKLR-17D1 has no effect on feeding. In summary, after food intake, the DSK neurons MP1/MP3 act on CCKLR-17D3 receptors in Gr64f-expressing GRNs to reduce sugar sensing and thereby diminish food-seeking and feeding (electronic supplementary material, figure S3B). The role of altered *takeout* expression in food-seeking remains to be determined.

The mechanisms by which DSK released from IPCs mediates satiety and reduced feeding [199] are not yet known. However, it could be speculated that the axon terminations of the IPCs on the crop release DSK that acts to influence crop muscle contractions and thereby inhibit further food intake, similar to mechanisms by which CCK induces satiety in mammals by affecting gastric emptying (see [207]). Another neuropeptide produced by brain neurons similar to the IPCs, *Drosophila* myosuppressin (DMS), has in fact been shown to act to modulate crop contractions and thereby increase food intake in female flies after mating [208].

Another role of DSK signalling in *Drosophila* is in regulation of aggression and courtship behaviour (figure 8, electronic supplementary material, figure S3C). It was shown that the MP1/MP3 neurons express Fru^M^ and are pre- and postsynaptic to the male-specific Fru^M^-expressing P1 neurons and hence are part of the circuitry regulating male-specific behaviour [88,89]. The P1 neurons form a heterogeneous cluster of about 20 neurons that integrate chemosensory, visual and mechanosensory cues from females to regulate sexual arousal, as well as cues from other males to control aggression [11,209–212]. This means that P1 neurons weigh diverse sensory inputs to control two opposing behaviours, courtship and aggression. The MP1/MP3 neurons are associated with P1 neurons in both behaviours, and DSK plus the two DSK receptors are important players in the behavioural modulation; CCKL-17D3 is targeted for modulation of sexual arousal and CCKL-17D1 for aggression [88,89] (figure 8; electronic supplementary material, figure S3C).

In male courtship behaviour, the P1 neurons are activated by appropriate sensory cues from females [11,209,210]. The P1 neurons act on descending neurons that activate courtship singing via pacemaker circuits in the thoracic neuromeres of the VNC [213]. Importantly, the courtship circuit is complex, as seen in electronic supplementary material, figure S3C, and involves further neurotransmitters and neuropeptides. The MP1/MP3 neurons receive inputs with cues about the external environment and the internal state such as housing conditions, metabolic state, and age, and depending on the valence of the sensory signals these neurons can inhibit the P1 neurons and thereby suppress courtship behaviour [89]. Moreover the MP1/MP3 neurons also suppress wakefulness and spontaneous walking [89]. It is worth noting that also in virgin females the MP1/MP3 neurons inhibit courtship behaviour by reducing the receptivity to courting male flies, but using different sex-specific circuitry (doublesex-expressing neurons) since female flies lack P1 neurons [89,214]. Thus, males and females alike use DSK neurons to interact with sex-dimorphic neurons to suppress courtship behaviour.

Male aggressive behaviour is controlled by circuitry that includes specific octopaminergic neurons (OANs), P1 neurons (a subpopulation of the sex-specific pC1 neurons) and a set of aSP2 neurons in the brain [215] (electronic supplementary material, figure S3C) and is modulated by dopamine, serotonin, TK, NPF and DSK [88,100,216]. The DSK-expressing MP1/MP3 neurons are part of the circuitry; being postsynaptic to the P1 neurons, they stimulate aggressive behaviour by acting on neurons expressing the CCKLR-17D1 receptor [88] (figure 8; electronic supplementary material, figure S3C). Hence the MP1/MP3 neurons are part of circuits controlling both courtship and aggression, and use two different DSK receptors to control these opposing behaviours. As seen in electronic supplementary material, figure S3C (inset), there is also a GABAergic circuit interconnecting P1 and pC1 neurons that form a switch between the two opposing behaviours (see [11]). It can be noted that the DSK-expressing IPCs have also been implicated in modulation of aggressive behaviour, but the downstream circuitry is not known [201,202].

### 5.4. Orchestrating signalling by hormones produced in the brain and corpora cardiaca

Centrally derived peptide hormones released into the circulation also mediate state-dependent signals to brain circuits and chemosensory cells in *Drosophila* and thereby modulate sensory inputs and behavioural outcomes, as well as physiology and metabolism. Thus, not only paracrine neuropeptides, but also peptide hormones can be included among the mediators of endocrine cybernetics. We focus here on AKH released from AKH-producing cells (APCs) in the corpora cardiaca and DILPs from brain IPCs, although there are several other peptide hormones that are produced by brain neurosecretory cells. These other hormones, which include peptides such as corazonin (CRZ), DH44, diuretic hormone 31 (DH31), and ITP that primarily regulate water and ion homeostasis and metabolism, as well as associated stress alleviation (see [18,120]), will not be dealt with here. We also ignore developmental functions of peptide hormones (see [18,120]). Instead, we briefly summarize some regulatory mechanisms that orchestrate behaviour in flies by means of hormonal signalling.

The *Drosophila* hormones AKH and DILPs display functions analogous to mammalian glucagon and insulin/insulin-like growth factors, respectively [174,217–221]. Although these hormones are commonly associated with regulation of metabolism in adult flies, they also affect nutrient-dependent behaviours (see [93,222–228]). Since DILP and AKH signalling ensures metabolic and energy homeostasis, the action of IPCs and APCs also have secondary effects in relation to most, if not all, behaviours. The metabolic homeostasis is tightly linked to nutrient-sensing, food-seeking and feeding, all of which involve DILP and AKH signalling. First, we discuss the regulatory roles of insulin signalling.

#### 5.4.1. Insulin signalling

We have already discussed (§5.1) the role of DILPs in nutrient state-dependent modulation of sensitivity of food odour sensing OSNs in the olfactory system by means of regulation of presynaptic expression of peptide GPCRs (figure 3*d*,*e*; electronic supplementary material, figure S1). This gain control mechanism mediates the state of hunger/satiety via DILP action in specific odour channels to increase food-seeking in hungry flies [107,108]. More directly, DILPs (inhibitory) and AKH (stimulatory) act on OANs that regulate exploratory activity and food search [228]. It was furthermore shown that the IPCs express a mechanosensory channel protein, Piezo, that monitors the crop distension via the IPC axon terminations on this organ and thereby generating satiety signals [229]. That study did not identify the target of the IPCs that inhibits feeding upon satiety, but excluded action of the DILPs 2, 3 and 5. Other signals were not tested. Maybe the IPCs use either DSK or a small molecule transmitter to target circuits in the SEG that control feeding, or possibly DSK is released from IPCs onto the crop to regulate its contractions, similar to DMS [208].

The IPCs receive clock inputs, are cell-autonomously nutrient-sensing and are known to partake in control of the fly's feeding rhythm [90,227,230] as well as its locomotor activity and sleep [226]. These cells also mediate metabolism-sleep interactions [93,231]. Starvation, specifically protein-deprivation, promotes loss of sleep and DILP2 signalling was found critical in regulation of starvation-induced sleep depth, thereby promoting amelioration of sleep loss [231]. Another study showed that sleep suppression in starved flies involves IPCs under control of inputs from the single pair of glucose-responsive LHLK neurons, which release LK during starvation [93].

Learning and memory functions are also influenced by DILP signalling. It was shown that the insulin receptor (dInR) substrate chico is expressed in the mushroom bodies and that memory formation in associative learning is defect in chico mutants [232]. Also, long-term memory is affected by genetic manipulations of dInR and Chico in the mushroom bodies [233]. Intermediate-term learning is dependent on DILP3 signalling to the fat body and the age-dependent impairment of memory appears to depend on loss of this signalling [234].

Finally, DILP signalling regulates courtship behaviour in flies. Female flies respond to the male attractant pheromone cis-vaccenyl acetate (cVA) in a nutrient-dependent fashion [235]. Knockdown of the dInR in the cVA-responsive antennal glomerulus, VM2, leads to diminished cVA attraction, suggesting that nutrient-dependent insulin signalling is part of the control of pheromone sensitivity in females. Male responses to cVA are also affected by DILP signalling [225]. In males, cVA acts as a deterrent for other males and thereby prevents male–male courtship. It was shown that overexpression of DILP2 in.IPCs increases male-male courtship [225]. The increased insulin signalling leads to promotion of juvenile hormone release from corpora allata which blocks ecdysone signalling. This causes downregulation of the cVA receptor Or67d in ORNs and reduced pheromone sensitivity, leading to increased courtship behaviour. A recent finding is that sugar intake by male flies increases insulin signalling which acts acutely on insulin receptors on Fru^M^-expressing P1 neurons and thereby suppresses male-to-female courtship [236]. The same pathway is also activated by repulsive pheromones via signalling from CCAP-expressing chemosensory cells to IPCs leading to decreased sexual activity when the male is exposed to suboptimal mates. The authors suggest that this mechanism ensures that the male flies make appropriate mating decisions when food availability fluctuates [236]. Taken together, insulin signalling appears to link nutritional state and behaviours such as food-seeking, activity and sleep, aggression, mating and conditioned learning and memory.

#### 5.4.2. AKH signalling

Apart from its metabolic functions in carbohydrate and lipid homeostasis, AKH is known to induce increased locomotor activity, indicative of food-seeking, in hungry flies [237,238]. As mentioned above AKH activates OANs to increase exploratory activity and food search [228]. Furthermore, in hungry flies, AKH stimulates a set of neurons called ISNs (interoceptive SEZ neurons) in the SEZ to increase sugar consumption [224]. By contrast, in fed flies, insulin signalling inhibits these ISNs. The same ISNs are sensitive to osmolarity via expression of the receptor nanchung and they decrease water intake at low osmolarity [224]. Thus, these AKH-responding cells regulate both drinking and sugar intake. In hungry flies AKH acts on bitter sensing Gr66a-expressing GRNs (possibly indirectly) to suppress their activity and thereby render flies less selective in their feeding [169]. AKH has also been found to increase contractions of the crop which might influence feeding [239].

An example of how AKH signalling regulates the competing behaviours activity and rest was shown recently. It was found that during the day AKH signalling via OANs increases activity in flies with access to food, whereas at night AKH acts on the fat body to decrease activity levels [240]. Finally, it was demonstrated that male flies with AKH receptor deficiency displayed decreased courtship activity when starved [222]. This is mediated by signalling to AKH receptor expressing SEZ neurons that innervate the antennal lobe, including glomeruli sensitive to the pheromone cVA [222]. Taken together it seems that AKH is involved in a number of nutrient-dependent behaviours, some of which are counter-regulated by DILPs.

### 5.5. Orchestrating signalling by hormones produced in enteroendocrine cells of the intestine

**Figure 9.**
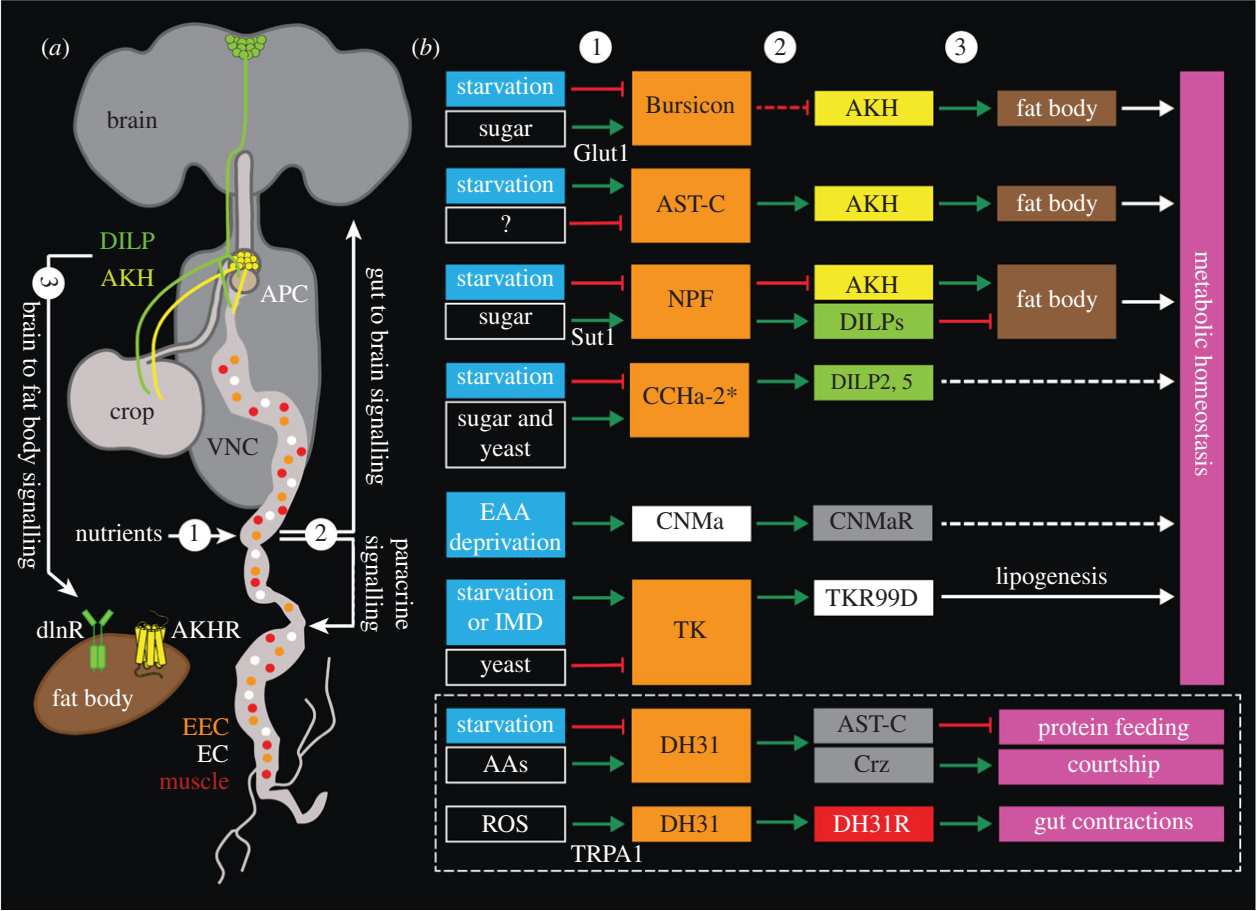
Interorgan peptide signalling: brain, intestine and fat body. Peptides from the gut modulate metabolic homeostasis, behaviours and gut physiology via state-dependent (mainly nutritional) gut-to-brain and paracrine signalling. See electronic supplementary material, figure S5 for distribution of gut peptides. (*a*) A schematic showing the signalling pathways mediating the effects of peptides produced by gut enteroendocrine cells (EECs; orange) and enterocytes (ECs; white). (1) These cells predominantly sense nutrients such as carbohydrates, yeast and amino acids, but they can also sense ROS, and receive inputs from the innate IMD pathway. (2) Once these cells are activated, they release peptides into the circulation for local effects (paracrine signalling) on gut enterocytes or muscles (red). In addition, the peptides target the nervous system, including the adipokinetic hormone (AKH)-producing cells (APCs) and *Drosophila* insulin-like peptide (DILP)-producing cells (IPCs). (3) AKH and DILPs in turn target their receptors (AKHR and dInR, respectively) on the fat body to influence metabolic homeostasis. (*b*) Various intestinal peptidergic pathways influence metabolic homeostasis in a feeding-state-dependent manner. Sugar activates Bursicon EECs via the Glut1 glucose transporter and Bursicon indirectly inhibits APCs to regulate lipid homeostasis [241]. Starvation triggers release of Allatostatin-C (AstC), which activates APCs to promote energy mobilization [242]. Neuropeptide F (NPF)-producing EECs respond to sugar via the Sut1 transporter, and released NPF inhibits APCs and activates IPCs to regulate lipid metabolism [243]. Sugars and yeast activate, while starvation inhibits release of CCHa-2 from cells in the gut and fat body. CCHa-2 stimulates release of DILP2 and 5 to influence organismal growth [244]. Deprivation of EAAs activates CNMa-producing ECs, to regulate metabolic homeostasis via actions on CNMaR-expressing neurons [245]. Both starvation and IMD pathway activate, whereas yeast inhibits tachykinin (TK)-producing EECs [161,162]. TK acts locally on enterocytes via its receptor TKR99D to affect lipogenesis. Amino acids activate diuretic hormone 31 (DH31) producing EECs [45]. DH31 regulates the balance between feeding and courtship via actions on AstC- and corazonin (CRZ)-producing neurons, respectively [39]. Moreover, DH31 EECs can also sense reactive oxygen species (ROS) via the TRPA1 receptor to regulate gut contractions [246]. Boxes in (*b*) are colour-coded to match the cell types in (*a*). Dashed arrows indicate indirect actions. The two receptors in the fat body were generated in BioRender.

The intestine is an ideal site for monitoring the nutritional state of an organism and mediating hormonal signals to the brain to adjust physiology and nutrient-dependent behaviour in order to maintain metabolic homeostasis. In *Drosophila* and other insects, such signals are primarily constituted by peptide hormones released from nutrient-sensing EECs of the intestine (figure 9) [32,221,247–250]. The *Drosophila* EECs and other gut cells produce at least 12 different peptides [32,40,41,245,250] (electronic supplementary material, figure S5). Some of these have been shown to act at a distance as circulating hormones targeting brain neurons or APCs in the corpora cardiaca. These hormones thereby mediate nutrient states sensed by the EECs to neurons and neurosecretory cells to trigger homeostatic responses (figure 9). As we shall see, some peptides affect behaviour more directly.

Sugar activates bursicon-producing EECs via the Glut1 glucose transporter, and the released bursicon indirectly inhibits APCs in the corpora cardiaca to regulate lipid homeostasis [241]. Starvation stimulates release of Allatostatin-C (AstC) from EECs, which activates AKH release to promote energy mobilization [242]. NPF-producing EECs express the Sut1 transporter, which mediates sugar sensing. Upon EEC activation NPF is released and acts on APCs to inhibit AKH release, and on IPCs to activate DILP release and thereby regulate lipid metabolism [243]. Sugars and yeast activate, while starvation inhibits the activity of CCHamide-2 (CCHa2) producing EECs in the gut and adipocytes in the fat body. CCHa2 stimulates release of DILP2 and DILP5 to influence organismal growth [244]. Deprivation of essential amino acids (EAAs) activates CNMamide (CNMa)-producing enterocytes, which affects metabolic homeostasis via actions on CNMa receptor (CNMaR) expressing neurons [245]. Both starvation and the immune deficiency (IMD) pathway activate, whereas yeast inhibits TK producing EECs [161,162]. The TK acts locally on enterocytes via its receptor TKR99D to affect lipogenesis. Moreover, DH31-producing EECs can also sense reactive oxygen species (ROS) via the TRPA1 receptor and release DH31 to regulate gut contractions [246]. This facilitates elimination of opportunistic bacteria. Gut peptides also influence behaviours more directly. Amino acids activate DH31-producing EECs [45]. The EEC-derived DH31 regulates the balance between feeding and courtship via actions on AstC- and CRZ-producing neurons, respectively [39].

Available data suggest that several gut-derived peptides converge onto the APC and IPCs. EEC-derived bursicon [241], AstC [242] and NPF [243] act on APCs, which in turn signal to the fat body to regulate metabolic homeostasis (figure 9). NPF [243] and CCHa2 [244] target brain IPCs to regulate DILP release for metabolic action opposing that of AKH (figure 9). AstA from EECs may additionally contribute to regulation of satiety via actions on IPCs and APCs [91]. Several of the regulatory mechanisms targeting APCs and IPCs affect behaviour via secondary hormones or neuromodulators, as outlined in the previous section (figure 9*b*).

## 6. Conclusion and future perspectives

We have outlined the organization of some key peptidergic systems that mediate state- and context-dependent regulation of behaviour in *Drosophila*. Peptidergic modulation is organized hierarchically with some key peptides at the top level having global orchestrating roles and others at a lower level with executive neuromodulatory functions in specific circuits. Executive modulation occurs already locally at the level of sensory cells to regulate their gain, but importantly this local neuromodulation is influenced by state-dependent higher-level signalling (see [2,8,10,12]). Characteristic for the higher-level systems is that they integrate multiple inputs that mediate cues from the external environment and internal states and subsequently regulate downstream circuits to ensure a relevant behavioural outcome. Concurrently, the same neuropeptides are involved in establishing metabolic and sleep homeostasis. Peptidergic systems mediate high-level neuromodulation by paracrine signalling [25,57,58,251] and thereby affect behavioural decisions and we refer to this signalling as endocrine cybernetics. The peptidergic systems discussed herein are only representative examples that highlight principles of how neuromodulation and behavioural plasticity is accomplished. Thus, our presentation is far from a comprehensive coverage of peptidergic signalling in the fly. For further examples we, therefore, refer to table 2, where we provide a comprehensive list of behaviours that are modulated by neuropeptides and peptide hormones. It is interesting to note that two of the peptidergic neuronal systems discussed here in more detail have been molecularly and functionally conserved over evolution; those using DSK and AstA. Thus, AstA is related to vertebrate galanin and these peptides regulate feeding, sleep and hormone release both in *Drosophila* and mammals (see [91,181]). Moreover, DSK and vertebrate CCK are ancestrally related and known to regulate satiety, feeding, aggression and reproductive behaviour in flies and mammals (see [207]).

**Table 2.** Neuropeptides regulating behaviours in *Drosophila*. This table is updated and altered from [18,35].

behaviour	neuropeptide	circuit/neurons	references
olfaction	sNPF	OSN-PN antennal lobe	[108]
	MIP	OSN-PN antennal lobe	[252]
		LN-PN	[253]
		antennal lobe	
	TK	LN-OSN-PN antennal lobe	[99,107]
	CCHa1	OSNs	[254]
		antennal lobe	
	DILPs	OSN-PN antennal lobe	[108]
		OSN (VM2)	[235]
	NPF	brain interneurons	[255,256]
	SIFa	global interneurons	[87]
taste	sNPF	LNCs-Gr66a^a^	[169,257]
	Hugin	hugin neurons (SEZ)	[194,258]
	DSK	MP1/MP3-Gr64f	[200]
	TK	pheromone pathway	[103]
	LK	LK neurons	[259]
	AKH	Gr5a	[224,228,260]
food search/feeding	NPF	brain interneurons	[92,261–267]
	sNPF	OSNs-PNs,	[20,108,168,261,268]
		local brain interneurons	
		MB circuits	
	TK	OSNs-PNs	[107]
		SMP-TK	[159]
	Hugin-PK	SEZ neurons	[258]
	sex peptide	via sperm in females	[59,269–272]
	DSK	IPCs, MP1/MP3	[199,200]
	DMS	PI neurons	[208]
	bursicon	EECs	[208]
	AKH	CC cells	[228,237,240,260,273]
	DILP8	tumour cells to brain neurons	[274]
	SIFa	global interneurons	[87]
	AstA	brain neurons or EECs	[20,91,180,181,275]
	AstC	EECs	[242]
	AstC	brain neurons	[39]
	CCHa2	gut, fat body	[244,276]
	DH44	MNCs and TAG	[96,277–280]
	DH31	EECs	[39]
	LK	brain neurons	[96,281]
	MIP	brain neurons	[282]
	ITP	LNCs	[102]
	CRZ	LNCs	[283]
thirst/drinking	AstA	JanuAstA	[92]
	NPF	brain AstA-R neurons	[92]
	ITP	ITPn/ALK	[102]
locomotion (independent of clock)	DSK	larvae	[284]
	proctolin	larvae	[285]
	PDF	outside clock	[286]
	AKH	CC larvae	[287]
	Ast-A	larvae	[275]
	LK	larvae	[288]
explorative walking	sNPF	central body	[98]
	TK	central body	[98]
clock/circadian activity/sleep	PDF	clock circuit	[165,289]
	sNPF	clock and other circuits	[165–167]
	NPF	clock circuit	[264,290,291]
	TK	fan-shaped body	[292,293]
	ITP	clock circuit	[294]
	DH31	clock circuit	[295,296]
	CCHa1	clock circuit	[297]
	sex peptide	in females	[298]
	LK	lateral horn neurons (LHLK)	[93,172,178,179]
	DH44	MNCs	[299–301]
	SIFa	global interneurons	[179,192,299]
	Hugin-PK	SEZ neurons	[300]
	MIP	brain neurons	[302]
	FMRFa	CNS neurons	[303]
	DILPs	IPCs	[301,304,305]
	bursicon	CNS neurons	[179]
	AstA	CNS neurons	[91,184]
	AstC	Clock circuit	[306]
	CNMamide	clock neurons	[307]
aggression^b^	NPF	brain interneurons	[216]
	sex peptide	in females	[308]
	DSK	IPCs	[201,202,309]
		MP1/MP3	[88]
	TK	LPP1B brain interneurons	[100]
	DH44	DH44-R1 cells^c^	[310]
	natalisin	brain interneurons	[155]
learning	sNPF	mushroom body	[164]
	corazonin	brain neurons^d^	[311]
	NPF	brain interneurons	[312–314]
	DILPs	IPCs	[232–234]
nociception	sNPF (DILP7)	interneurons^e^	[170]
	TK	interneurons^f^	[109,315]
	Ast-C	Ast-C receptor	[316]
	LK	LK receptor	[317]
		LK neurons^g^	[318]
	DILP7	interneurons^e^	[318]
ethanol-related behaviours	corazonin	LNCs	[319,320]
	DILPs	IPCs	[321]
	NPF	brain interneurons	[322]
courtship	NPF	brain interneurons	[257,322,323]
	corazonin	TAG	[311,324]
		brain neurons	[39]
	DH44	MNCs	[325]
	PDF	clock circuit	[257,326]
	sex peptide	sperm transfer	[59,327–329]
	SIFa	global interneurons	[186,191]
	natalisin	brain interneurons	[155]
	MIP	interneurons TAG (females)	[330,331]
	DSK	MP1/MP3	[89]
	DILPs	IPCs	[225,235,236]
sperm storage	DH44	in females	[325]
egg laying	DILP7	TAG efferents	[332]

^a^The LNCs regulate Gr66 bitter taste with sNPF.

^b^Males if not indicated.

^c^Specific cells in circuit not known.

^d^A successful copulation is a reward in male flies and strengthens long-term appetitive memories.

^e^Interneurons in thoracico-abdominal ganglion of larvae.

^f^Neurosecretory cells in thoracico-abdominal ganglion of larvae.

Abbreviations: Gr5a, Gr64f and Gr66a, gustatory receptors; IPCs, insulin-producing cells; JanuAstA, AstA-expressing interneurons; LN, local neurons; LNCs, lateral neurosecretory cells; MB, mushroom bodies; MNCs, median neurosecretory cells; MP1/MP3, DSK-expressing interneurons, OSN, olfactory sensory neurons; PN, projection neurons; SEZ, suboesophageal zone; SMP-TK, TAG, thoracico-abdominal ganglion; TK expressing interneurons; VM2, pheromone-sensitive glomerulus in antennal lobe.

Behaviours are supervised by anatomically defined synaptic circuits in brain centres, like the central complex and mushroom bodies, and groups of interconnected neurons such as the sex-specific P1/pC1 neurons and clock neurons. These circuits include smaller or larger sets of intrinsic peptidergic neurons, but also receive modulatory inputs from extrinsic peptidergic neurons. Since peptidergic neuromodulation is predominantly non-synaptic it has so far not been possible to establish a complete ‘peptidergic connectome’ in the fly. With analysis of conventional synapses, it is possible to determine how identified peptidergic neurons connect to other neurons, but the resulting connectome is likely to predominantly represent signalling with SMNs. Thus, the connections of a set of identified peptidergic neurosecretory cells and associated neurons in the brain of the early *Drosophila* larva [194,333], and a partial connectome of the adult clock neurons, many of which are peptidergic [334,335] tell us little about peptidergic signalling. Yet, these studies are vital since they inform us that peptidergic neurons do signal quite extensively also with SMNs. In the clock system, this is especially interesting since data on non-peptidergic signalling is scarce (see [336–338]).

**Figure 10.**
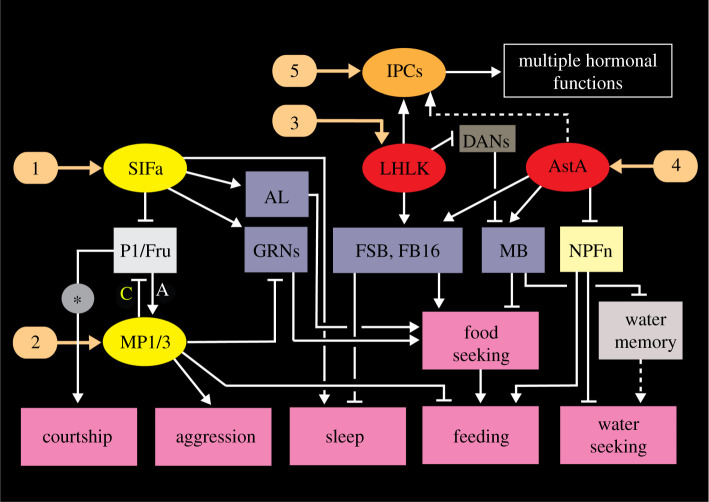
Summary diagram of peptide signalling that regulates competing behaviours. SIFamide (SIFa) and drosulfakinin (DSK)-expressing (MP1/3) neurons interact with several brain circuits to regulate behaviours in an orchestrating fashion, some of which are competing. One hub that regulates courtship versus aggression under peptidergic influence is a set of Fru^M^-expressing P1 neurons. The asterisk indicates additional neuronal circuitry (see electronic supplementary material, figure S3). SIFa and DSK also target antennal lobe (AL) neurons and gustatory receptor neurons (GRNs) to regulate food search and feeding. LHLKs are leucokinin-expressing neurons that target different circuits in the fan-shaped body (FSB in general, or layer 16, FBl6, specifically) or mushroom bodies (MB) via dopaminergic neurons (DANs) to regulate sleep and food choice. LHLKs also regulate insulin-producing cells (IPCs) to affect sleep/metabolism interactions. Allatostatin-A neurons (AstA) target FSB and MB to regulate food choice and sleep, and neuropeptide F-expressing neurons (NPFn) to modulate feeding and water seeking. The IPCs release insulin-like peptides (DILPs) to regulate feeding, metabolism, sleep and other functions. The numbers (1–5) indicate inputs to the different neurons systems as follows, 1 (SIFa): myoinhibitory peptide, hugin-PK, clock inputs, and probably corazonin (CRZ), DILPs, DSK and short neuropeptide F (sNPF); 2 (MP1/MP3): nutritional state, P1 neurons, age, housing conditions; 3 (LHLK): nutritional state, thirst, clock; 4 (AstA): dopamine, pigment-dispersing factor, nutritional state; 5 (IPCs) nutritional state, clock, GABA, serotonin, octopamine, dopamine, DILPs, allatostatin-C (AstC), neuropeptide F (NPF), short NPF (sNPF), CCHamide2, tachykinin (TK), adipokinetic hormone (AKH), and CRZ. For references, see text.

We show here that different peptidergic systems in *Drosophila* interact with the circuits of the central complex, mushroom bodies and P1/pC1 neurons in context-dependent regulation of behaviour. The peptidergic interactions with these systems in regulation of courtship, aggression, sleep/wake, food-seeking, feeding and water seeking are summarized in figure 10. Each of the key peptidergic neurons shown (using SIFa, DSK, LK, AstA and DILPs) receives multiple inputs that regulate their activity dependent on internal and external cues. Thus, a complex set of interactions ensures an appropriate behavioural outcome based on weighing different inputs. In electronic supplementary material, figure S6, we show a simple scheme illustrating how these peptidergic cells converge on different behaviours in their regulatory capacities. This figure indicates that food search and feeding is regulated by all the peptidergic systems listed above (using SIFa, DSK, LK, AstA and DILPs), sleep/wake is regulated by all, but the DSK neurons, whereas aggression only by DSK (and maybe DILPs). It can be noted that these behaviours are also regulated at some level by multiple other neuropeptides shown in electronic supplementary material, figure S6 and also in table 2. The hierarchical organization of peptidergic modulatory systems probably allows for a greater behavioural plasticity since there are several more points/levels of intervention. Furthermore, combining parallel and hierarchical modulatory systems, each with inputs mediating different external and internal cues, ensures appropriate behavioural decisions.

Several other peptidergic systems, that were ignored here, are of interest for analysis of context-dependent neuromodulation of neuronal circuits at the level between orchestrating and executive neuromodulation. Thus, not only LK and AstA-expressing neurons, but also ones signalling with AstC, CRZ, DH31, DH44, Hugin-pyrokinin, myoinhibitory peptide, natalisin and NPF seem to be intermediate size neurons with multiple inputs and in a position to modulate circuits that affect behaviour (table 2 and references therein). For analysis of executive neuromodulation by neuropeptides and co-transmission with SMNs, the antennal lobe/olfactory system and the clock circuits are excellent established models, especially since many of the intrinsic neurons are peptidergic [85,116–118,335], and their connectomes are available [75,334,339].

How can we proceed to unravel more complex interactions between neuronal circuits controlling behaviour where neuropeptides are involved? Connectomics studies in *Drosophila* has revealed an immense complexity of synaptic circuits in brain, and the data acquired so far has generated testable models for neuronal mechanisms in learning and memory formation, in visual navigation, as well as in visual and olfactory signal processing [73–76]. However, much work remains to translate physical connections to real-time circuit function and neuronal plasticity in the behaving fly. The temporal dynamics and the excitatory or inhibitory nature of the synapses needs to be established and the synaptic transmitters and properties of their cognate receptors remain to be determined for each neuron. As emphasized in this review, and by others, another important aspect needed for a more complete understanding of brain circuits is the contribution of local and global neuromodulation [22,23,72,340]. To delineate the spatio-temperal role of paracrine signalling in the CNS, one needs to map the relevant peptide/monoamine receptors in ‘target neurons' in relation to the ‘sender neurons', and monitor how far the neuromodulator can diffuse within the brain. The distance travelled by a peptide neuromodulator is limited by dilution, diffusion barriers and by the distribution of membrane-bound proteolytic enzymes that inactivate the neuropeptides [25,57,341–343]. Thus, determination of a whole set of parameters is required that cannot be visualized from analysis of serial section electron microscopy, but require alternative strategies. For large-scale analysis of a peptidergic ‘connectome’, new genetic approaches are required to complement anterograde [344,345] and retrograde [346] transsynaptic labelling techniques. Genetic sensors to monitor peptidergic modulation *in vivo* could partly fill this gap [347–349]. However, these sensors need further optimization to be suitable for large-scale peptidergic connectome analyses. In addition, spatially mapped single-cell transcriptomics could also be used to map the distribution of cells/neurons producing the neuropeptides (source) and the cells expressing the receptors (target), as has been used in the marine annelid *Platynereis dumerilii* [350]. While high expression of neuropeptides and relatively low expression of neuropeptide GPCRs makes this approach challenging at present (due to false positives and false negatives, respectively) [351], advances in sequencing technologies and increasing the number of cells analysed will surely improve this strategy in the future.

In the meantime, experimental analysis with genetic tools continues to provide important information about the modulatory roles of neuropeptides in different behaviours. Further mapping of peptide GPCRs is definitely needed, especially with the goal to locate receptor protein and establishing receptor function. We also need to take into account the pleiotropic functions of neuropeptides (see [35]) and that several neuropeptides can converge onto the same brain circuit. Most neuropeptides discovered in insects seem to be functionally pleiotropic, but some have not yet been investigated in enough detail to disclose their functional repertoire (see [18,35]). At any rate, it is likely that quite a number of the neuropeptides each act in several different circuits to mediate various modulatory functions; some of these may be functionally independent as described for TKs in *Drosophila* (figure 3) [106]. Early on, insect neuropeptides were assigned functions based on single assays and even received names reflecting these first discovered functions, such as PDF, natalisin, diuretic hormones and allatostatins. Today, it is apparent that these functional names can be misleading since the peptide either has no such function in the insect in question, or while multiple novel functions have been discovered. The latter means that it is not always clear what is the primary function of a neuropeptide (if any). Thus, the functional analysis of a neuropeptide mutant may reveal a behaviour change resulting from a conglomerate of effects at different levels of the brain. This means that further analysis needs to address the peptide function in each circuit level.

Another feature that has emerged over recent years is that multiple peptidergic systems converge onto specific circuits and thus the regulation of the same behaviours (or physiology). Here, we have shown this in the case of regulation of food search and feeding, as well as courtship, aggression and sleep (figures 6 and 10; electronic supplementary material, figure S6). Several neuropeptides and peptide hormones additionally converge onto pathways that regulate development, memory formation and metabolic and water/ion homeostasis (figures 4, 6 and 9). Probably this multiplicity in peptidergic systems regulating a specific circuit reflects how different adaptive contexts (internal states and external stimuli) influence behaviour/physiology by means of separate peptidergic pathways. The different peptidergic signals converging on a specific behaviour are not necessarily redundant, but likely to operate under specific contexts and/or timescales. Probably we need to shift our thinking from trying to identify a single function for a given neuropeptide to uncovering a parsimonious explanation for the multiple functions of a given peptide. Such functions may nevertheless enable the animal to make behavioural decisions and thus the neuropeptide partakes in endocrine cybernetics.

## Data Availability

This article has no additional data.
